# Data on optimized production and characterization of alkaline proteases from newly isolated alkaliphiles from Lonar soda lake, India

**DOI:** 10.1016/j.dib.2016.06.044

**Published:** 2016-07-05

**Authors:** Mukundraj Govindrao Rathod, Anupama Prabhakarrao Pathak

**Affiliations:** School of Life Sciences, S.R.T.M. University, Nanded, Maharashtra, India

**Keywords:** Alkaline protease, Optimization, Lonar lake, Polyphasic

## Abstract

Alkaline proteases are one of the industrially important enzymes and generally preferred from alkaliphilic sources. Here we have provided the data on optimized production and characterization of alkaline proteases from five newly isolated and identified alkaliphiles from Lonar soda lake, India. The data provided for optimization of physicochemical parameters for maximum alkaline proteases production is based on OVAT (one variable at a time) approach. Alkaline protease production (U/mL) recorded by using different agro industrial residues is included in the given data. Further readers can find more information in our previously published research article where we have already described about the methods used and comparative analysis of the data recorded regarding optimized production, characterization and application of alkaline proteases isolated from Lonar soda lake isolates (http://dx.doi.org/10.1016/j.bcab.2016.06.002) [1]. The data provided here by us is useful to other researchers for setting up various suitable statistical models to perform optimization studies other than OVAT approach.

**Specifications Table**

**Table:** 

Subject area	*Biology*
More specific subject area	*Biocatalysis and Industrial biotechnology*
Type of data	*Tables*
How data was acquired	*Orbital shaking incubator* (*Remi make, Vasai, Mumbai, Model no. CIS-24 BL*) *was used to perform optimization of physicochemical parameters for maximum alkaline protease production. The instruments used to perform alkaline protease activity assay were cooling centrifuge machine* (*Remi, Mumbai*)*, and UV double beam spectrophotometer* (*Shimadzu corporation*)
Data format	*Raw and analyzed*
Experimental factors	*We isolated and further identified by polyphasic approach*[1], [2], [3], [4], [5], [6], [7], [8], [9], [10], [11], [15]*five alkaline protease producers namely Brachybacterium* sp. LAP214, *Bacillus cohnii* LAP217, *Bacillus pseudofirmus* LAP220, *Brevibacterium casei* LAP223 and *Halomonas venusta* LAP515 from Lonar soda lake, India. Selected experimental factors to optimize alkaline protease production were pH, temperature, incubation period, carbon sources, nitrogen sources and inducers in OVAT (one variable at a time) approach. For alkaline protease characterization, the experimental factors selected were pH, temperatures, substrates, activators, inhibitors, metal cations, chelator, surfactants and oxidizing agents
Experimental features	*Total protein contents were determined using bovine serum albumin as standard*[12]. *Alkaline protease activities were determined by the modified Anson׳s method as described by Yang and Wang*[13]. *One unit of protease activity was defined as the amount of the enzyme that releases* 1 µmol/mL/min *of tyrosine equivalent under the assay conditions. All experiments were performed in triplicates and average values were calculated. Further*, *standard deviations (n*=3*) were calculated to understand experimental errors caused. The analyses of data were performed by using MS-Excel 2013 software*
Data source location	*Lonar soda lake, India* (19°59′N, 76°31′E)
Data accessibility	*Data is within this article and cultures are available at the Microbial Culture Collection, NCCS, Pune, India under the accession numbers* MCC 2834, MCC 2819, MCC 2820, MCC 2890 and MCC 2955 at the link http://www.nccs.res.in/mcc/Bacteria.html[14]. 16S rRNA partial gene sequences of these isolates are available under the accession numbers GenBank: KP995734, GenBank: KP995735, GenBank: KP995736 and KP995737, and GenBank: KR186012 at http://www.ncbi.nlm.nih.gov/genbank/

**Value of the data**

• The data provided by us help to understand the effect of each factor exerted at a time.
• The data provided by us is useful to other researchers for setting up various suitable statistical models for optimization studies.
• The given data shows quantity of alkaline proteases produced by using different agro-industrial residues.
• The given data is important to study catalytic behavior of alkaline proteases in presence of selected range of pH, temperature, substrate concentrations, activators, inhibitors, metal cations, chelator, surfactants and oxidizing agents.
• The given data can also be used for its representation into graphical forms.

## 1 Data

The tabular data presented here contain the values of optical densities (absorbance at 600 nm) and alkaline protease production (U/mL) recorded while assessing the effect of physical parameters like pH, temperature, incubation period, agitation speed etc. (Table 1–15 and Table 21–25) and chemical parameters like carbon and nitrogen sources, inducers etc. (Table 16–20 and Table 26–45) on growth and production. Furthermore, the data contain values of enzyme activities (U/mL) with standard deviation (*n*=3) and their relative activities (%) recorded in presence of different substrates and at various pH and temperatures (Table 46–65). Moreover the data presented here contain the values of enzyme activities (U/mL) and residual activities (%) recorded in presence of selected activators, inhibitors, metal cations and commercial detergents at their varying concentrations (Table 66–85).

## 2 Experimental design, materials and methods

### 2.1 Optimization of physicochemical parameters, production, partial purification and characterization of alkaline proteases

Methods adopted for optimization of physicochemical parameters, production, partial purification and characterization of alkaline proteases from afore-mentioned isolates have been already described by us in our previously published article (http://dx.doi.org/10.1016/j.bcab.2016.06.002) [1].
