# Supplementary material for: Data on optimized production and characterization of alkaline proteases from newly isolated alkaliphiles from Lonar soda lake, India
Source: Data Brief. 2016 Jul 5;8:863–6. doi: 10.1016/j.dib.2016.06.044 (PMC4957540; doi:10.1016/j.dib.2016.06.044)
Supplement: Supplementary file 2 — Supplementary material [file mmc2.doc]

**Table 1:** Effect of time coarse and agitation speed on production of alkaline protease from *Halomonas venusta* LAP515

| Isolate  → | *Halomonas venusta* LAP515 | | | | | | | |
| --- | --- | --- | --- | --- | --- | --- | --- | --- |
| Time period→  Agitation speed (rpm)↓ | 24 h | | 48 h | | 72 h | | 96 h | |
|  | A.P. | S.D. | A.P. | S.D. | A.P. | S.D. | A.P. | S.D. |
| 0 | 78.47 | 4.65 | 123.67 | 2.87 | 306.45 | 6.84 | 350.54 | 5.98 |
| 50 | 123.56 | 2.67 | 141.87 | 3.76 | 349.34 | 5.97 | 356.87 | 4.87 |
| **100** | 136.34 | 5.87 | 264.34 | 4.87 | **420.12** | 6.04 | 400.34 | 3.98 |
| 150 | 140.76 | 3.87 | 257.65 | 5.85 | 400.45 | 7.96 | 390.56 | 7.95 |
| 200 | 135.67 | 4.76 | 240.76 | 7.83 | 390.57 | 3.87 | 310.87 | 5.94 |

**Table 2:** Effect of time coarse and agitation speed on production of alkaline protease from *Brachybacterium* sp. LAP214

| Isolate  → | *Brachybacterium* sp. LAP214 | | | | | | | |
| --- | --- | --- | --- | --- | --- | --- | --- | --- |
| Time period→  Agitation speed (rpm)↓ | 24 h | | 48 h | | 72 h | | 96 h | |
|  | A.P. | S.D. | A.P. | S.D. | A.P. | S.D. | A.P. | S.D. |
| 0 | 34.98 | 3.54 | 210.75 | 2.65 | 239.87 | 3.65 | 240.76 | 2.56 |
| 50 | 108.83 | 5.35 | 221.84 | 4.65 | 250.76 | 3.87 | 270.94 | 4.76 |
| 100 | 120.56 | 2.65 | 225.84 | 3.98 | 310.65 | 4.76 | 300.84 | 4.65 |
| **150** | 150.38 | 6.25 | 249.85 | 4.63 | **340.76** | 3.65 | 314.95 | 2.65 |
| 200 | 142.73 | 3.87 | 233.85 | 2.56 | 330.85 | 2.65 | 311.74 | 4.87 |

Abbreviations: A.P. = Alkaline protease production (U/mL); S.D. = Standard Deviation (n=3)

**Table 3:** Effect of time coarse and agitation speed on production of alkaline protease from *Bacillus pseudofirmus* LAP220

| Isolate  → | *Bacillus pseudofirmus* LAP220 | | | | | | | |
| --- | --- | --- | --- | --- | --- | --- | --- | --- |
| Time period→  Agitation speed (rpm)↓ | 24 h | | **48 h** | | 72 h | | 96 h | |
|  | A.P. | S.D. | A.P. | S.D. | A.P. | S.D. | A.P. | S.D. |
| 0 | 51.73 | 2.65 | 102.83 | 4.63 | 90.84 | 2.65 | 88.63 | 5.76 |
| 50 | 67.38 | 3.87 | 154.83 | 2.76 | 112.34 | 4.76 | 110.64 | 3.25 |
| **100** | 90.84 | 4.87 | **311.47** | 3.87 | 290.85 | 4.25 | 288.47 | 2.76 |
| 150 | 70.94 | 3.85 | 218.78 | 4.62 | 200.85 | 3.87 | 191.97 | 4.67 |
| 200 | 67.74 | 4.86 | 211.83 | 3.65 | 198.48 | 4.89 | 142.84 | 3.26 |

**Table 4:** Effect of time coarse and agitation speed on production of alkaline protease from *Bacillus cohnii* LAP217

| Isolate  → | *Bacillus cohnii* LAP217 | | | | | | | |
| --- | --- | --- | --- | --- | --- | --- | --- | --- |
| Time period→  Agitation speed (rpm)↓ | 24 h | | **48 h** | | 72 h | | 96 h | |
|  | A.P. | S.D. | A.P. | S.D. | A.P. | S.D. | A.P. | S.D. |
| 0 | 90.82 | 4.93 | 210.84 | 2.65 | 200.72 | 3.76 | 190.83 | 3.76 |
| 50 | 103.72 | 3.76 | 266.89 | 3.65 | 255.38 | 3.76 | 240.82 | 3.75 |
| **100** | 210.84 | 5.87 | **347.87** | 3.76 | 312.94 | 4.62 | 300.85 | 2.67 |
| 150 | 198.73 | 5.34 | 310.83 | 2.67 | 294.38 | 2.75 | 291.83 | 1.87 |
| 200 | 191.93 | 3.76 | 300.82 | 4.65 | 291.83 | 1.87 | 288.84 | 3.87 |

**Table 5:** Effect of time coarse and agitation speed on production of alkaline protease from *Brevibacterium casei* LAP223

| Isolate  → | *Brevibacterium casei* LAP223 | | | | | | | |
| --- | --- | --- | --- | --- | --- | --- | --- | --- |
| Time period→  Agitation speed (rpm)↓ | 24 h | | 48 h | | **72 h** | | 96 h | |
|  | A.P. | S.D. | A.P. | S.D. | A.P. | S.D. | A.P. | S.D. |
| 0 | 106.63 | 2.76 | 201.74 | 3.85 | 256.94 | 3.73 | 251.87 | 3.86 |
| 50 | 143.82 | 3.96 | 231.84 | 4.83 | 260.84 | 2.86 | 255.81 | 3.82 |
| 100 | 168.17 | 4.83 | 260.83 | 4.76 | 319.95 | 5.84 | 306.84 | 4.26 |
| **150** | 201.43 | 6.84 | 272.75 | 1.85 | **340.74** | 5.83 | 335.83 | 1.85 |
| 200 | 190.36 | 5.38 | 265.74 | 3.96 | 320.85 | 2.85 | 301.74 | 6.37 |

Abbreviations: A.P. = Alkaline protease production (U/mL); S.D. = Standard Deviation (n=3)

**Table 6:** Effect of pH on growth and production of alkaline protease from *Halomonas venusta* LAP515

| Isolate → | *Halomonas venusta* LAP515 | | | |
| --- | --- | --- | --- | --- |
| pH↓ | Growth | | Alkaline protease production | |
|  | A_600_ | S.D. | U/mL | S.D. |
| 8 | 0.4873 | 0.0034 | 90.75 | 3.85 |
| 9 | 0.9763 | 0.0087 | 398.92 | 4.83 |
| **10** | **1.9959** | 0.0024 | **420.12** | 6.04 |
| 11 | 0.5621 | 0.0031 | 215.87 | 5.83 |

SD=standard deviation (n=3)

**Table 7:** Effect of pH on growth and production of alkaline protease from *Brachybacterium* sp. LAP214

| Isolate → | *Brachybacterium* sp. LAP214 | | | |
| --- | --- | --- | --- | --- |
| pH↓ | Growth | | Alkaline protease production | |
|  | A_600_ | S.D. | U/mL | S.D. |
| 8 | 0.4832 | 0.0043 | 115.83 | 5.82 |
| 9 | 0.9654 | 0.0065 | 263.93 | 4.23 |
| **10** | **2.5432** | 0.0053 | **340.76** | 3.65 |
| 11 | 1.4539 | 0.0025 | 310.93 | 2.93 |

S.D. = Standard Deviation (n=3)

**Table 8:** Effect of pH on growth and production of alkaline protease from *Bacillus pseudofirmus* LAP220

| Isolate → | *Bacillus pseudofirmus* LAP220 | | | |
| --- | --- | --- | --- | --- |
| pH↓ | Growth | | Alkaline protease production | |
|  | A_600_ | S.D. | U/mL | S.D. |
| 8 | 0.5875 | 0.0045 | 228.84 | 4.73 |
| 9 | 1.5874 | 0.0054 | 276.74 | 3.76 |
| **10** | **2.5092** | **0.0031** | **311.47** | 3.87 |
| 11 | 0.5174 | 0.0065 | 298.87 | 5.43 |

**Table 9:** Effect of pH on growth and production of alkaline protease from *Bacillus cohnii* LAP217

| Isolate → | *Bacillus cohnii* LAP217 | | | |
| --- | --- | --- | --- | --- |
| pH↓ | Growth | | Alkaline protease production | |
|  | A_600_ | S.D. | U/mL | S.D. |
| 8 | 0.7685 | 0.0054 | 209.83 | 4.83 |
| 9 | 1.8746 | 0.0035 | 267.57 | 5.93 |
| **10** | **2.3762** | 0.0021 | **347.87** | 3.76 |
| 11 | 0.9563 | 0.0074 | 309.74 | 4.38 |

**Table 10:** Effect of pH on growth and production of alkaline protease from *Brevibacterium casei* LAP223

| Isolate → | *Brevibacterium casei* LAP223 | | | |
| --- | --- | --- | --- | --- |
| pH↓ | Growth | | Alkaline protease production | |
|  | A_600_ | S.D. | U/mL | S.D. |
| 8 | 0.7864 | 0.0042 | 204.83 | 6.34 |
| 9 | 1.7432 | 0.0218 | 302.72 | 4.38 |
| **10** | **2.0842** | 0.0321 | **340.74** | 5.83 |
| 11 | 0.9874 | 0.0142 | 310.37 | 7.32 |

S.D. = Standard Deviation (n=3)

**Table 11:** Effect of temperature on growth and production of alkaline protease from *Halomonas venusta* LAP515

| Isolate → | *Halomonas venusta* LAP515 | | | |
| --- | --- | --- | --- | --- |
| Temperature ^o^C↓ | Growth | | Alkaline protease production | |
|  | A_600_ | S.D. | U/mL | S.D. |
| 20 | **1.9973** | 0.0032 | 411.83 | 4.39 |
| **30** | 1.9423 | 0.0067 | **420.12** | 6.04 |
| 40 | 1.6532 | 0.0062 | 372.38 | 5.38 |

**Table 12:** Effect of temperature on growth and production of alkaline protease from *Brachybacterium* sp. LAP214

| Isolate → | *Brachybacterium* sp. LAP214 | | | |
| --- | --- | --- | --- | --- |
| Temperature ^o^C↓ | Growth | | Alkaline protease production | |
|  | A_600_ | S.D. | U/mL | S.D. |
| 20 | 1.6634 | 0.0323 | 103.93 | 5.38 |
| **30** | **2.5432** | 0.0053 | **340.76** | 3.65 |
| 40 | 0.6762 | 0.0245 | 242.94 | 4.39 |

S.D. = Standard Deviation (n=3)

**Table 13:** Effect of temperature on growth and production of alkaline protease from *Bacillus pseudofirmus* LAP220

| Isolate → | *Bacillus pseudofirmus* LAP220 | | | |
| --- | --- | --- | --- | --- |
| Temperature ^o^C↓ | Growth | | Alkaline protease production | |
|  | A_600_ | S.D. | U/mL | S.D. |
| 20 | 1.9324 | 0.0043 | 284.38 | 4.35 |
| **30** | **2.5092** | 0.0031 | **311.47** | 3.87 |
| 40 | 2.1932 | 0.0193 | 302.43 | 6.43 |

**Table 14:** Effect of temperature on growth and production of alkaline protease from *Bacillus cohnii* LAP217

| Isolate → | *Bacillus cohnii* LAP217 | | | |
| --- | --- | --- | --- | --- |
| Temperature ^o^C↓ | Growth | | Alkaline protease production | |
|  | A_600_ | S.D. | U/mL | S.D. |
| 20 | 1.8733 | 0.0047 | 304.93 | 4.93 |
| **30** | **2.3762** | 0.0021 | **347.87** | 3.76 |
| 40 | 1.6343 | 0.0183 | 320.84 | 5.82 |

**Table 15:** Effect of temperature on growth and production of alkaline protease from *Brevibacterium casei* LAP223

| Isolate → | *Brevibacterium casei* LAP223 | | | |
| --- | --- | --- | --- | --- |
| Temperature ^o^C↓ | Growth | | Alkaline protease production | |
|  | A_600_ | S.D. | U/mL | S.D. |
| 20 | 1.3321 | 0.0072 | 318.83 | 4.92 |
| **30** | **2.0842** | 0.0321 | **340.74** | 5.83 |
| 40 | 1.6732 | 0.0079 | 298.71 | 7.82 |

S.D. = Standard Deviation (n=3)

**Table 16:** Effect of NaCl on growth and production of alkaline protease from *Halomonas venusta* LAP515

| Isolate → | *Halomonas venusta* LAP515 | | | |
| --- | --- | --- | --- | --- |
| NaCl (%) ↓ | Growth | | Alkaline protease production | |
|  | A_600_ | S.D. | U/mL | S.D. |
| 0 | 1.9423 | 0.0067 | 420.12 | 6.04 |
| **1** | **2.0163** | 0.0024 | **502.92** | 5.38 |
| 2 | 1.9479 | 0.0025 | 410.83 | 5.93 |
| 3 | 1.9173 | 0.0173 | 398.93 | 4.93 |
| 4 | 1.8742 | 0.0045 | 374.85 | 7.91 |
| 5 | 1.0934 | 0.0072 | 310.88 | 1.93 |
| 6 | 0.8924 | 0.0047 | 274.94 | 5.92 |
| 7 | 0.3824 | 0.0016 | 105.48 | 9.84 |

**Table 17:** Effect of NaCl on growth and production of alkaline protease from *Brachybacterium* sp. LAP214

| Isolate → | *Brachybacterium* sp. LAP214 | | | |
| --- | --- | --- | --- | --- |
| NaCl (%) ↓ | Growth | | Alkaline protease production | |
|  | A_600_ | S.D. | U/mL | S.D. |
| 0 | 2.5432 | 0.0053 | 340.76 | 3.65 |
| **1** | **2.7521** | 0.0184 | **590.82** | 4.92 |
| 2 | 2.3823 | 0.0735 | 511.94 | 5.92 |
| 3 | 2.1943 | 0.0422 | 492.92 | 7.93 |
| 4 | 1.9835 | 0.0053 | 395.90 | 6.21 |
| 5 | 1.7463 | 0.0059 | 321.94 | 5.04 |
| 6 | 1.0723 | 0.0063 | 296.92 | 8.92 |
| 7 | 0.8234 | 0.0058 | 198.83 | 8.43 |

S.D. = Standard Deviation (n=3)

**Table 18:** Effect of NaCl on growth and production of alkaline protease from *Bacillus pseudofirmus* LAP220

| Isolate → | *Bacillus pseudofirmus* LAP220 | | | |
| --- | --- | --- | --- | --- |
| NaCl (%) ↓ | Growth | | Alkaline protease production | |
|  | A_600_ | S.D. | U/mL | S.D. |
| 0 | 2.5092 | 0.0031 | 311.47 | 3.87 |
| **1** | **2.6721** | 0.0054 | **452.65** | 5.82 |
| 2 | 2.4832 | 0.0014 | 451.64 | 4.99 |
| 3 | 2.1452 | 0.0053 | 490.35 | 5.83 |
| 4 | 1.8436 | 0.0064 | 352.65 | 6.04 |
| 5 | 1.6432 | 0.0026 | 298.54 | 5.93 |
| 6 | 1.2955 | 0.0075 | 201.54 | 1.95 |
| 7 | 0.9425 | 0.0093 | 198.04 | 5.93 |

**Table 19:** Effect of NaCl on growth and production of alkaline protease from *Bacillus cohnii* LAP217

| Isolate → | *Bacillus cohnii* LAP217 | | | |
| --- | --- | --- | --- | --- |
| NaCl (%) ↓ | Growth | | Alkaline protease production | |
|  | A_600_ | S.D. | U/mL | S.D. |
| 0 | 2.3762 | 0.0021 | 347.87 | 3.76 |
| **1** | **2.4823** | 0.0073 | **502.84** | 4.93 |
| 2 | 2.1834 | 0.0028 | 493.83 | 5.94 |
| 3 | 1.9849 | 0.0074 | 411.94 | 6.93 |
| 4 | 1.6734 | 0.0053 | 397.93 | 3.92 |
| 5 | 1.0341 | 0.0072 | 294.92 | 5.85 |
| 6 | 0.9943 | 0.0075 | 138.94 | 6.84 |
| 7 | 0.7641 | 0.0184 | 119.24 | 7.71 |

**Table 20:** Effect of NaCl on growth and production of alkaline protease from *Brevibacterium casei* LAP223

| Isolate → | *Brevibacterium casei* LAP223 | | | |
| --- | --- | --- | --- | --- |
| NaCl (%) ↓ | Growth | | Alkaline protease production | |
|  | A_600_ | S.D. | U/mL | S.D. |
| 0 | 2.0842 | 0.0321 | 340.74 | 5.83 |
| **1** | **2.2745** | 0.0533 | **582.94** | 6.83 |
| 2 | 2.1932 | 0.0015 | 523.95 | 9.85 |
| 3 | 1.9843 | 0.0036 | 426.90 | 4.83 |
| 4 | 1.6744 | 0.0064 | 419.95 | 3.87 |
| 5 | 1.0834 | 0.0036 | 398.91 | 7.95 |
| 6 | 0.9823 | 0.0073 | 284.94 | 5.93 |
| 7 | 0.5743 | 0.0038 | 88.13 | 6.29 |

**Table 21:** Effect of inoculum size on growth and production of alkaline protease from *Halomonas venusta* LAP515

| Isolate → | *Halomonas venusta* LAP515 | | | |
| --- | --- | --- | --- | --- |
| Inoculum volume (%) ↓ | Growth | | Alkaline protease production | |
|  | A_600_ | S.D. | U/mL | S.D. |
| 1 | 2.0163 | 0.0024 | 502.92 | 5.38 |
| 2 | 2.0579 | 0.0076 | 694.83 | 8.94 |
| 3 | 2.5833 | 0.0174 | 788.25 | 4.93 |
| **4** | **2.7643** | 0.0198 | **946.84** | 6.93 |
| 5 | 2.4344 | 0.0109 | 835.83 | 5.28 |

**Table 22:** Effect of inoculum size on growth and production of alkaline protease from *Brachybacterium* sp. LAP214

| Isolate → | *Brachybacterium* sp. LAP214 | | | |
| --- | --- | --- | --- | --- |
| Inoculum volume (%) ↓ | Growth | | Alkaline protease production | |
|  | A_600_ | S.D. | U/mL | S.D. |
| 1 | 2.7521 | 0.0184 | 590.82 | 4.92 |
| **2** | **2.9843** | 0.0638 | **873.87** | 5.93 |
| 3 | 2.9746 | 0.0274 | 783.92 | 6.93 |
| 4 | 2.8743 | 0.0073 | 627.93 | 5.94 |
| 5 | 2.8173 | 0.0124 | 583.85 | 7.64 |

S.D. = Standard Deviation (n=3)

**Table 23:** Effect of inoculum size on growth and production of alkaline protease from *Bacillus pseudofirmus* LAP220

| Isolate → | *Bacillus pseudofirmus* LAP220 | | | |
| --- | --- | --- | --- | --- |
| Inoculum volume (%) ↓ | Growth | | Alkaline protease production | |
|  | A_600_ | S.D. | U/mL | S.D. |
| 1 | 2.6721 | 0.0054 | 452.65 | 5.82 |
| 2 | 2.6846 | 0.0073 | 583.92 | 9.84 |
| **3** | **2.8946** | 0.0025 | **786.86** | 4.83 |
| 4 | 2.8783 | 0.0026 | 629.84 | 6.82 |
| 5 | 2.8184 | 0.0072 | 518.47 | 4.85 |

**Table 24:** Effect of inoculum size on growth and production of alkaline protease from *Bacillus cohnii* LAP217

| Isolate → | *Bacillus cohnii* LAP217 | | | |
| --- | --- | --- | --- | --- |
| Inoculum volume (%) ↓ | Growth | | Alkaline protease production | |
|  | A_600_ | S.D. | U/mL | S.D. |
| 1 | 2.4823 | 0.0073 | 502.84 | 4.93 |
| 2 | 2.6724 | 0.0071 | 793.92 | 5.93 |
| **3** | **2.9847** | 0.0084 | **895.47** | 5.72 |
| 4 | 2.8745 | 0.0063 | 812.84 | 6.94 |
| 5 | 2.8084 | 0.0042 | 801.93 | 9.84 |

**Table 25:** Effect of inoculum size on growth and production of alkaline protease from *Brevibacterium casei* LAP223

| Isolate → | *Brevibacterium casei* LAP223 | | | |
| --- | --- | --- | --- | --- |
| Inoculum volume (%) ↓ | Growth | | Alkaline protease production | |
|  | A_600_ | S.D. | U/mL | S.D. |
| 1 | 2.2745 | 0.0533 | 582.94 | 6.83 |
| 2 | 2.3742 | 0.0074 | 643.87 | 3.85 |
| 3 | 2.5109 | 0.0053 | 795.54 | 5.93 |
| **4** | **2.9841** | 0.0061 | **921.75** | 6.94 |
| 5 | 2.8746 | 0.0064 | 902.87 | 3.82 |

S.D. = Standard Deviation (n=3)

**Table 26:** Effect of selected inducers on growth and production of alkaline protease from *Halomonas venusta* LAP515

| Isolate → | *Halomonas venusta* LAP515 | | | |
| --- | --- | --- | --- | --- |
| Inducers ↓ | Growth | | Alkaline protease production | |
|  | A_600_ | S.D. | U/mL | S.D. |
| **Skimmed milk** | **2.9847** | 0.0083 | **1336.95** | 4.92 |
| Casein | 2.7643 | 0.0198 | 946.84 | 6.93 |
| Soybean seed flour | 2.7630 | 0.0073 | 942.97 | 4.93 |
| Defatted seed meal of soybean | 2.7643 | 0.0062 | 940.92 | 5.92 |
| Pumpkin seeds flour | 2.8635 | 0.0093 | 935.61 | 7.48 |
| Feather meal | 2.7646 | 0.0047 | 931.73 | 6.29 |
| Gelatin | 2.6583 | 0.0017 | 927.63 | 7.99 |
| Defatted seed meal of sunflower | 2.4872 | 0.0063 | 921.63 | 2.84 |
| Casein acid hydrolysate | 2.2875 | 0.0062 | 920.64 | 8.02 |
| Green Gram seed flour | 2.6483 | 0.0084 | 917.94 | 5.93 |
| Defatted seed meal of corn | 2.6332 | 0.0061 | 900.73 | 1.03 |
| Yeast extract | 2.7642 | 0.0063 | 863.98 | 4.93 |
| Dried powder of cow dung | 2.0946 | 0.0062 | 860.83 | 4.11 |
| Chick pea seeds flour | 2.1375 | 0.0071 | 851.94 | 3.29 |
| Defatted seed meal of groundnut | 2.6473 | 0.0053 | 845.92 | 4.93 |
| Meat extract | 2.4836 | 0.0064 | 841.93 | 5.04 |
| Defatted seed meal of safflower | 2.4082 | 0.0063 | 836.66 | 5.83 |
| Seed flour of corn | 2.1938 | 0.0187 | 831.51 | 2.92 |
| Beef extract | 2.1830 | 0.0284 | 826.33 | 3.98 |
| Peptone | 2.1193 | 0.0174 | 821.31 | 4.93 |
| Defatted seed meal of sesame | 2.0982 | 0.0524 | 813.82 | 5.83 |
| Tryptone | 2.0674 | 0.0073 | 714.81 | 4.91 |
| Defatted seed meal of cotton | 2.0678 | 0.0024 | 373.89 | 5.62 |
| Defatted seed meal of mustard | 1.9374 | 0.0083 | 182.83 | 4.98 |

S.D. = Standard Deviation (n=3)

**Table 27:** Effect of selected inducers on growth and production of alkaline protease from *Brachybacterium* sp. LAP214

| Isolate → | *Brachybacterium* sp. LAP214 | | | |
| --- | --- | --- | --- | --- |
| Inducers ↓ | Growth | | Alkaline protease production | |
|  | A_600_ | S.D. | U/mL | S.D. |
| **Soybean seed flour** | **3.1837** | 0.0075 | **4573.83** | 4.83 |
| Skimmed milk | 3.1753 | 0.0018 | 3985.13 | 5.83 |
| Defatted seed meal of soybean | 3.0723 | 0.0173 | 2847.84 | 7.93 |
| Casein | 2.9843 | 0.0638 | 873.87 | 5.93 |
| Defatted seed meal of safflower | 2.9809 | 0.0143 | 870.83 | 7.46 |
| Casein acid hydrolysate | 2.9283 | 0.0260 | 862.72 | 3.82 |
| Gelatin | 2.8763 | 0.0150 | 860.18 | 7.93 |
| Pumpkin seeds flour | 2.8644 | 0.0224 | 851.83 | 2.98 |
| Defatted seed meal of sunflower | 2.8173 | 0.0568 | 717.38 | 4.85 |
| Chick pea seeds flour | 2.7973 | 0.0034 | 628.92 | 2.83 |
| Feather meal | 2.7635 | 0.0132 | 512.94 | 4.93 |
| Seed flour of corn | 2.7520 | 0.0011 | 508.90 | 5.84 |
| Dried powder of cow dung | 2.7493 | 0.0027 | 501.93 | 5.34 |
| Yeast extract | 2.7193 | 0.0027 | 499.91 | 7.29 |
| Defatted seed meal of groundnut | 2.6432 | 0.0028 | 473.88 | 6.27 |
| Green Gram seed flour | 2.8932 | 0.0016 | 471.83 | 4.83 |
| Defatted seed meal of corn | 2.7482 | 0.0054 | 455.93 | 6.47 |
| Meat extract | 2.9831 | 0.0065 | 451.83 | 5.19 |
| Defatted seed meal of cotton | 2.6534 | 0.0027 | 442.93 | 4.83 |
| Defatted seed meal of sesame | 2.0544 | 0.0025 | 441.91 | 7.63 |
| Beef extract | 2.9830 | 0.0087 | 439.29 | 5.71 |
| Tryptone | 2.7633 | 0.0026 | 431.83 | 6.94 |
| Peptone | 2.1922 | 0.0028 | 420.83 | 5.03 |
| Defatted seed meal of mustard | 1.8733 | 0.0018 | 411.83 | 7.83 |

S.D. = Standard Deviation (n=3)

**Table 28:** Effect of selected inducers on growth and production of alkaline protease from *Bacillus pseudofirmus* LAP220

| Isolate → | *Bacillus pseudofirmus* LAP220 | | | |
| --- | --- | --- | --- | --- |
| Inducers ↓ | Growth | | Alkaline protease production | |
|  | A_600_ | S.D. | U/mL | S.D. |
| **Defatted seed meal of soybean** | **2.9876** | 0.0054 | **4072.82** | 2.84 |
| Soybean seed flour | 2.9811 | 0.0076 | 3982.93 | 3.76 |
| Skimmed milk | 2.9793 | 0.0028 | 3173.86 | 4.61 |
| Gelatin | 2.9766 | 0.0174 | 2733.33 | 8.47 |
| Casein | 2.8946 | 0.0025 | 786.86 | 4.83 |
| Casein acid hydrolysate | 2.7635 | 0.0382 | 780.26 | 9.64 |
| Feather meal | 2.7563 | 0.0472 | 771.36 | 7.46 |
| Defatted seed meal of sunflower | 2.7522 | 0.0058 | 765.93 | 8.63 |
| Green Gram seed flour | 2.7493 | 0.0042 | 761.37 | 9.63 |
| Defatted seed meal of groundnut | 2.7362 | 0.0082 | 750.27 | 6.01 |
| Chick pea seeds flour | 2.7183 | 0.0011 | 701.38 | 4.87 |
| Pumpkin seeds flour | 2.8736 | 0.0039 | 684.39 | 8.36 |
| Dried powder of cow dung | 2.7531 | 0.0284 | 672.31 | 9.27 |
| Defatted seed meal of safflower | 2.6541 | 0.0018 | 670.28 | 7.04 |
| Defatted seed meal of corn | 2.6073 | 0.0036 | 663.98 | 6.94 |
| Yeast extract | 2.5844 | 0.0063 | 661.28 | 4.93 |
| Peptone | 2.5064 | 0.0037 | 654.92 | 6.28 |
| Tryptone | 2.4928 | 0.0067 | 651.28 | 9.38 |
| Defatted seed meal of cotton | 2.3826 | 0.0042 | 642.83 | 2.84 |
| Meat extract | 2.2647 | 0.0047 | 636.38 | 7.47 |
| Seed flour of corn | 2.6742 | 0.0027 | 633.26 | 4.83 |
| Beef extract | 2.9842 | 0.0023 | 601.34 | 8.28 |
| Defatted seed meal of sesame | 2.3842 | 0.0074 | 598.26 | 4.87 |
| Defatted seed meal of mustard | 1.0033 | 0.0024 | 203.27 | 3.81 |

S.D. = Standard Deviation (n=3)

**Table 29:** Effect of selected inducers on growth and production of alkaline protease from *Bacillus cohnii* LAP217

| Isolate → | *Bacillus cohnii* LAP217 | | | |
| --- | --- | --- | --- | --- |
| Inducers ↓ | Growth | | Alkaline protease production | |
|  | A_600_ | S.D. | U/mL | S.D. |
| **Pumpkin seeds flour** | **3.1533** | 0.0047 | **3872.34** | 4.83 |
| Casein | 2.9847 | 0.0084 | 895.47 | 5.72 |
| Skimmed milk | 3.0924 | 0.0073 | 890.24 | 8.37 |
| Feather meal | 3.0093 | 0.0062 | 880.27 | 6.27 |
| Gelatin | 2.9873 | 0.0076 | 872.73 | 4.38 |
| Defatted seed meal of soybean | 2.9802 | 0.1730 | 871.93 | 5.83 |
| Casein acid hydrolysate | 2.9763 | 0.0369 | 860.35 | 7.93 |
| Defatted seed meal of sunflower | 2.9183 | 0.0047 | 855.38 | 7.27 |
| Soybean seed flour | 2.8633 | 0.0054 | 851.49 | 7.46 |
| Dried powder of cow dung | 2.8364 | 0.0027 | 840.93 | 8.28 |
| Green Gram seed flour | 2.8473 | 0.0016 | 837.93 | 9.84 |
| Defatted seed meal of corn | 2.8632 | 0.0074 | 831.29 | 10.84 |
| Defatted seed meal of safflower | 2.8725 | 0.0037 | 825.83 | 7.37 |
| Chick pea seeds flour | 2.8563 | 0.0087 | 821.75 | 4.38 |
| Defatted seed meal of groundnut | 2.8463 | 0.0036 | 815.38 | 9.74 |
| Peptone | 2.8137 | 0.0033 | 800.35 | 6.38 |
| Tryptone | 2.7638 | 0.0014 | 743.39 | 5.96 |
| Yeast extract | 2.8339 | 0.0026 | 741.38 | 2.98 |
| Meat extract | 2.7163 | 0.0076 | 735.38 | 4.87 |
| Defatted seed meal of cotton | 2.6738 | 0.0054 | 721.39 | 9.15 |
| Seed flour of corn | 2.8739 | 0.0087 | 714.39 | 6.05 |
| Beef extract | 2.7732 | 0.0026 | 702.18 | 3.05 |
| Defatted seed meal of sesame | 2.0932 | 0.0027 | 683.28 | 2.96 |
| Defatted seed meal of mustard | 1.0086 | 0.0084 | 680.26 | 1.12 |

S.D. = Standard Deviation (n=3)

**Table 30:** Effect of selected inducers on growth and production of alkaline protease from *Brevibacterium casei* LAP223

| Isolate → | *Brevibacterium casei* LAP223 | | | |
| --- | --- | --- | --- | --- |
| Inducers ↓ | Growth | | Alkaline protease production | |
|  | A_600_ | S.D. | U/mL | S.D. |
| **Soybean seed flour** | **3.8742** | 0.0037 | **3873.97** | 3.92 |
| Skimmed milk | 3.8173 | 0.0042 | 3093.83 | 4.38 |
| Chick pea seeds flour | 3.8072 | 0.0157 | 2873.48 | 5.28 |
| Gelatin | 3.7183 | 0.0046 | 2193.72 | 5.39 |
| Casein acid hydrolysate | 3.2835 | 0.0026 | 1093.37 | 9.73 |
| Casein | 2.9841 | 0.0061 | 921.75 | 6.94 |
| Pumpkin seeds flour | 2.9085 | 0.0047 | 917.28 | 10.28 |
| Defatted seed meal of soybean | 2.8472 | 0.0014 | 915.37 | 9.27 |
| Feather meal | 2.8174 | 0.0133 | 911.34 | 7.38 |
| Defatted seed meal of groundnut | 2.8084 | 0.1830 | 909.38 | 6.04 |
| Green Gram seed flour | 2.7382 | 0.0143 | 890.37 | 9.09 |
| Dried powder of cow dung | 2.7134 | 0.0143 | 883.82 | 7.01 |
| Yeast extract | 2.7052 | 0.0024 | 871.28 | 2.21 |
| Seed flour of corn | 2.6936 | 0.0037 | 860.21 | 4.87 |
| Defatted seed meal of sesame | 2.6436 | 0.0073 | 851.28 | 6.25 |
| Defatted seed meal of cotton | 2.6143 | 0.0072 | 843.29 | 9.27 |
| Defatted seed meal of corn | 2.5942 | 0.0173 | 841.28 | 9.04 |
| Defatted seed meal of sunflower | 2.5475 | 0.0384 | 830.21 | 4.83 |
| Defatted seed meal of safflower | 2.5137 | 0.0038 | 825.72 | 1.28 |
| Peptone | 2.5064 | 0.0037 | 821.27 | 4.38 |
| Beef extract | 2.4965 | 0.0024 | 811.26 | 5.95 |
| Meat extract | 2.4675 | 0.0021 | 810.21 | 3.09 |
| Tryptone | 2.4146 | 0.0034 | 802.28 | 8.43 |
| Defatted seed meal of mustard | 1.0395 | 0.0023 | 539.29 | 3.09 |

S.D. = Standard Deviation (n=3)

**Table 31:** Effect of different concentration of skimmed milk on growth and production of alkaline protease from *Halomonas venusta* LAP515

| Isolate → | *Halomonas venusta* LAP515 | | | |
| --- | --- | --- | --- | --- |
| Skimmed milk (%) ↓ | Growth | | Alkaline protease production | |
|  | A_600_ | S.D. | U/mL | S.D. |
| 0.5 | 1.8732 | 0.0024 | 754.93 | 3.95 |
| 1.0 | 2.9847 | 0.0083 | 1336.95 | 4.92 |
| 1.5 | 2.9983 | 0.0273 | 2128.28 | 5.49 |
| 2.0 | 3.3232 | 0.0024 | 2903.21 | 7.38 |
| **2.5** | **3.3562** | 0.0084 | **3092.83** | 5.49 |
| 3.0 | 3.3763 | 0.0093 | 2983.48 | 7.31 |

S.D. = Standard Deviation (n=3)

**Table 32:** Effect of different concentration of soybean seed flour on growth and production of alkaline protease from *Brachybacterium* sp. LAP214

| Isolate → | *Brachybacterium* sp. LAP214 | | | |
| --- | --- | --- | --- | --- |
| Soybean seed flour (%) ↓ | Growth | | Alkaline protease production | |
|  | A_600_ | S.D. | U/mL | S.D. |
| 0.5 | 1.9837 | 0.1324 | 1093.39 | 5.49 |
| 1.0 | 3.1837 | 0.0075 | 4573.83 | 4.83 |
| 1.5 | 3.2872 | 0.0173 | 4673.29 | 4.92 |
| **2.0** | **3.3723** | 0.0024 | **4893.23** | 7.27 |
| 2.5 | 3.3452 | 0.0021 | 4563.21 | 4.39 |
| 3.0 | 3.3273 | 0.0073 | 4493.18 | 5.21 |

S.D. = Standard Deviation (n=3)

**Table 33:** Effect of different concentration of defatted seed meal of soybean on growth and production of alkaline protease from *Bacillus pseudofirmus* LAP220

| Isolate → | *Bacillus pseudofirmus* LAP220 | | | |
| --- | --- | --- | --- | --- |
| Defatted seed meal of soybean  (%) ↓ | Growth | | Alkaline protease production | |
|  | A_600_ | S.D. | U/mL | S.D. |
| 0.5 | 1.8372 | 0.0065 | 2083.28 | 3.98 |
| 1.0 | 2.9876 | 0.0054 | 4072.82 | 2.84 |
| 1.5 | 2.9921 | 0.0037 | 4183.39 | 3.27 |
| 2.0 | 3.1311 | 0.0016 | 4383.87 | 4.92 |
| **2.5** | **3.4360** | 0.0027 | **4598.28** | 4.38 |
| 3.0 | 3.3142 | 0.0045 | 4203.39 | 5.39 |

**Table 34:** Effect of different concentration of pumpkin seed flour on growth and production of alkaline protease from *Bacillus cohnii* LAP217

| Isolate → | *Bacillus cohnii* LAP217 | | | |
| --- | --- | --- | --- | --- |
| Flour of pumpkin seeds  (%) ↓ | Growth | | Alkaline protease production | |
|  | A_600_ | S.D. | U/mL | S.D. |
| 0.5 | 1.9832 | 0.0038 | 1074.38 | 12.07 |
| 1.0 | 3.1533 | 0.0047 | 3872.34 | 4.83 |
| 1.5 | 3.1837 | 0.0032 | 3984.23 | 4.48 |
| **2.0** | **3.2837** | 0.0183 | **4039.39** | 11.39 |
| 2.5 | 3.2983 | 0.0284 | 3874.21 | 4.73 |
| 3.0 | 3.3183 | 0.0021 | 3533.49 | 5.49 |

**Table 35:** Effect of different concentration of soybean seed flour on growth and production of alkaline protease from *Brevibacterium casei* LAP223

| Isolate → | *Brevibacterium casei* LAP223 | | | |
| --- | --- | --- | --- | --- |
| Flour of soybean seeds  (%) ↓ | Growth | | Alkaline protease production | |
|  | A_600_ | S.D. | U/mL | S.D. |
| 0.5 | 2.1831 | 0.0013 | 1483.34 | 5.48 |
| 1.0 | 3.8742 | 0.0037 | 3873.97 | 3.92 |
| **1.5** | **3.9324** | 0.0017 | **4056.28** | 7.28 |
| 2.0 | 3.8372 | 0.0043 | 3987.29 | 8.39 |
| 2.5 | 3.2343 | 0.0014 | 3193.28 | 9.37 |
| 3.0 | 3.1028 | 0.0019 | 3093.65 | 4.38 |

S.D. = Standard Deviation (n=3)

**Table 36:** Effect of different carbon and nitrogen sources on growth and production of alkaline protease from *Halomonas venusta* LAP515

| Isolate → | *Halomonas venusta* LAP515 | | | |
| --- | --- | --- | --- | --- |
| Carbon and nitrogen sources  (1 %) ↓ | Growth | | Alkaline protease production | |
|  | A_600_ | ± S.D. | U/mL | ± S.D. |
| **Sucrose** | **3.6833** | 0.0083 | **5092.83** | 4.93 |
| Glucose | 3.6109 | 0.0038 | 5027.38 | 4.91 |
| Lactose | 3.3590 | 0.0184 | 3091.36 | 4.97 |
| Mannitol | 3.3261 | 0.0073 | 4873.22 | 5.49 |
| Sugarcane molasses | 3.3052 | 0.0283 | 4725.27 | 7.93 |
| Sorbitol | 3.3034 | 0.0047 | 4625.39 | 9.26 |
| Ribose | 3.3193 | 0.0124 | 4572.21 | 10.38 |
| Sugarcane bagasse | 3.3229 | 0.0382 | 4482.98 | 12.38 |
| Wheat bran | 3.4634 | 0.0012 | 4416.39 | 11.39 |
| Glycerol | 3.4724 | 0.0372 | 4400.03 | 2.03 |
| Xylose | 3.4837 | 0.0356 | 4376.08 | 6.93 |
| Rice bran | 3.5642 | 0.0023 | 4316.93 | 13.39 |
| Arabinose | 3.4837 | 0.0038 | 4274.91 | 9.03 |
| **Ammonium chloride** | **3.7526** | 0.0042 | **6524.92** | 6.49 |
| Ammonium sulphate | 3.7142 | 0.0124 | 6153.39 | 9.03 |
| Ammonium nitrate | 3.7032 | 0.0638 | 6083.19 | 10.39 |

**Table 37:** Effect of different carbon and nitrogen sources on growth and production of alkaline protease from *Brachybacterium* sp. LAP214

| Isolate → | *Brachybacterium* sp. LAP214 | | | |
| --- | --- | --- | --- | --- |
| Carbon and nitrogen sources (1 %) ↓ | Growth | | Alkaline protease production | |
|  | A_600_ | ± S.D. | U/mL | ± S.D. |
| **Glucose** | **3.5837** | 0.0024 | **5093.94** | 7.27 |
| Xylose | 3.5804 | 0.0073 | 5003.27 | 6.48 |
| Mannitol | 3.4139 | 0.0027 | 4891.38 | 5.39 |
| Arabinose | 3.3253 | 0.0072 | 4890.23 | 7.28 |
| Glycerol | 3.3167 | 0.0055 | 4866.29 | 5.93 |
| Sugarcane bagasse | 3.3094 | 0.0043 | 4862.63 | 7.27 |
| Lactose | 3.2842 | 0.0073 | 4851.39 | 5.49 |
| Sugarcane molasses | 3.1834 | 0.0017 | 4850.92 | 5.39 |
| Sucrose | 3.0942 | 0.0035 | 4848.94 | 5.29 |
| Sorbitol | 3.1290 | 0.0037 | 4844.42 | 9.05 |
| Ribose | 3.1403 | 0.0062 | 4839.27 | 9.23 |
| Wheat bran | 3.1102 | 0.0037 | 4839.21 | 11.39 |
| Rice bran | 3.1232 | 0.0033 | 4824.27 | 5.94 |
| **Ammonium chloride** | **3.5892** | 0.0043 | **5588.37** | 8.04 |
| Ammonium sulphate | 3.5023 | 0.0027 | 5482.45 | 5.94 |
| Ammonium nitrate | 3.4830 | 0.0072 | 5133.60 | 6.12 |

**Table 38:** Effect of different carbon and nitrogen sources on growth and production of alkaline protease from *Bacillus pseudofirmus* LAP220

| Isolate → | *Bacillus pseudofirmus* LAP220 | | | |
| --- | --- | --- | --- | --- |
| Carbon and nitrogen sources  (1 %) ↓ | Growth | | Alkaline protease production | |
|  | A_600_ | ± S.D. | U/mL | ± S.D. |
| **Glucose** | **3.4360** | 0.0027 | **4598.28** | 4.38 |
| Sucrose | 3.3722 | 0.0173 | 4591.84 | 5.93 |
| Sugarcane molasses | 3.3242 | 0.0037 | 4543.42 | 7.91 |
| Lactose | 3.3153 | 0.0037 | 4540.41 | 9.03 |
| Arabinose | 3.3150 | 0.0071 | 4535.13 | 5.93 |
| Mannitol | 3.3076 | 0.0054 | 4532.24 | 7.34 |
| Sorbitol | 3.3071 | 0.0004 | 4531.49 | 8.73 |
| Ribose | 3.3040 | 0.0003 | 4529.44 | 6.02 |
| Sugarcane bagasse | 3.3013 | 0.0036 | 4528.45 | 9.04 |
| Rice bran | 3.3012 | 0.0027 | 4533.35 | 5.48 |
| Wheat bran | 3.2945 | 0.0017 | 4431.35 | 4.38 |
| Glycerol | 3.2856 | 0.0273 | 4415.32 | 7.37 |
| Xylose | 3.2805 | 0.0183 | 4414.31 | 5.31 |
| **Ammonium sulphate** | **3.4893** | 0.0102 | **4893.39** | 3.09 |
| Ammonium chloride | 3.4794 | 0.0123 | 4734.25 | 6.44 |
| Ammonium nitrate | 3.4173 | 0.0223 | 4618.39 | 5.48 |

**Table 39:** Effect of different carbon and nitrogen sources on growth and production of alkaline protease from *Bacillus cohnii* LAP217

| Isolate → | *Bacillus cohnii* LAP217 | | | |
| --- | --- | --- | --- | --- |
| Carbon and nitrogen sources  (1 %) ↓ | Growth | | Alkaline protease production | |
|  | A_600_ | ± S.D. | U/mL | ± S.D. |
| **Glucose** | **3.3627** | 0.0183 | **5251.36** | 11.39 |
| Lactose | 3.2821 | 0.0181 | 4035.27 | 4.38 |
| Sugarcane molasses | 3.2811 | 0.0243 | 4031.27 | 4.82 |
| Sucrose | 3.2809 | 0.0047 | 4025.72 | 5.49 |
| Mannitol | 3.2805 | 0.0017 | 4024.27 | 6.34 |
| Sorbitol | 3.2801 | 0.0027 | 4021.27 | 11.37 |
| Ribose | 3.2787 | 0.0037 | 4019.34 | 17.38 |
| Sugarcane bagasse | 3.2781 | 0.0063 | 4012.83 | 11.37 |
| Rice bran | 3.2750 | 0.0046 | 4011.36 | 9.37 |
| Wheat bran | 3.2813 | 0.0031 | 4009.31 | 6.48 |
| Glycerol | 3.2803 | 0.0036 | 4001.75 | 13.28 |
| Arabinose | 3.2812 | 0.0063 | 3912.85 | 2.00 |
| Xylose | 3.2735 | 0.0137 | 3910.64 | 4.39 |
| **Ammonium chloride** | **3.3829** | 0.0033 | **5837.38** | 7.28 |
| Ammonium sulphate | 3.3772 | 0.0043 | 5802.26 | 9.47 |
| Ammonium nitrate | 3.3163 | 0.0065 | 5783.87 | 4.38 |

**Table 40:** Effect of different carbon and nitrogen sources on growth and production of alkaline protease from *Brevibacterium casei* LAP223

| Isolate → | *Brevibacterium casei* LAP223 | | | |
| --- | --- | --- | --- | --- |
| Carbon and nitrogen sources  (1 %) ↓ | Growth | | Alkaline protease production | |
|  | A_600_ | ± S.D. | U/mL | ± S.D. |
| **Lactose** | **3.9736** | 0.0017 | **5833.27** | 7.28 |
| Glucose | 3.9711 | 0.0073 | 5732.15 | 4.65 |
| Sucrose | 3.9700 | 0.0042 | 5711.27 | 7.34 |
| Sugarcane molasses | 3.9173 | 0.0024 | 4090.27 | 8.15 |
| Mannitol | 3.9145 | 0.0014 | 4056.26 | 6.36 |
| Sugarcane bagasse | 3.9017 | 0.0076 | 4051.33 | 7.45 |
| Sorbitol | 3.8763 | 0.0074 | 4047.18 | 7.23 |
| Ribose | 3.8637 | 0.0064 | 4044.31 | 6.15 |
| Rice bran | 3.8632 | 0.0065 | 4041.36 | 7.35 |
| Wheat bran | 3.8611 | 0.0154 | 4039.18 | 5.11 |
| Xylose | 3.8542 | 0.0270 | 4031.36 | 9.34 |
| Arabinose | 3.8531 | 0.0014 | 4028.28 | 21.45 |
| Glycerol | 3.8511 | 0.0022 | 4021.23 | 11.34 |
| **Ammonium chloride** | **3.9867** | 0.0024 | **5974.85** | 9.35 |
| Ammonium sulphate | 3.9736 | 0.0042 | 5831.83 | 5.24 |
| Ammonium nitrate | 3.9711 | 0.0016 | 5824.81 | 11.45 |

**Table 41:** Effect of different concentrations of sucrose and ammonium chloride on production of alkaline protease from *Halomonas venusta* LAP515

| Isolate → | *Halomonas venusta* LAP515 | |
| --- | --- | --- |
| Sucrose (%) ↓ | Alkaline protease production | |
|  | U/mL | ± S.D. |
| **0.5** | **6735.38** | 6.83 |
| 1.0 | 5092.83 | 4.93 |
| 1.5 | 5019.52 | 9.37 |
| 2.0 | 5003.64 | 5.43 |
| Ammonium chloride (%) ↓ | | |
| 0.5 | 6032.38 | 5.77 |
| 1.0 | 6524.92 | 6.49 |
| **1.5** | **6932.27** | 4.99 |
| 2.0 | 6662.08 | 3.27 |

S.D. = Standard Deviation (n=3)

**Table 42:** Effect of different concentrations of glucose and ammonium chloride on production of alkaline protease from *Brachybacterium* sp. LAP214

| Isolate → | *Brachybacterium* sp. LAP214 | |
| --- | --- | --- |
| Glucose (%) ↓ | Alkaline protease production | |
|  | U/mL | ± S.D. |
| 0.5 | 4983.32 | 3.29 |
| **1.0** | **5093.94** | 7.27 |
| 1.5 | 4991.24 | 7.33 |
| 2.0 | 4833.23 | 4.38 |
| Ammonium chloride (%) ↓ | | |
| 0.5 | 5231.93 | 7.38 |
| 1.0 | 5588.37 | 8.04 |
| **1.5** | **7832.34** | 7.24 |
| 2.0 | 6722.09 | 8.46 |

**Table 43:** Effect of different concentrations of glucose and ammonium sulphate on production of alkaline protease from *Bacillus pseudofirmus* LAP220

| Isolate → | *Bacillus pseudofirmus* LAP220 | |
| --- | --- | --- |
| Glucose (%) ↓ | Alkaline protease production | |
|  | U/mL | ± S.D. |
| 0.5 | 4432.98 | 5.48 |
| 1.0 | 4598.28 | 4.38 |
| **1.5** | **6434.32** | 9.24 |
| 2.0 | 6183.26 | 4.26 |
| Ammonium sulphate (%) ↓ | | |
| **0.5** | **7192.23** | 6.49 |
| 1.0 | 4893.39 | 3.09 |
| 1.5 | 4172.28 | 8.46 |
| 2.0 | 4092.37 | 9.41 |

S.D. = Standard Deviation (n=3)

**Table 44:** Effect of different concentrations of glucose and ammonium chloride on production of alkaline protease from *Bacillus cohnii* LAP217

| Isolate → | *Bacillus cohnii* LAP217 | |
| --- | --- | --- |
| Glucose (%) ↓ | Alkaline protease production | |
|  | U/mL | ± S.D. |
| **0.5** | **6543.24** | 3.56 |
| 1.0 | 5251.36 | 11.39 |
| 1.5 | 5183.09 | 4.33 |
| 2.0 | 5023.29 | 6.44 |
| Ammonium chloride (%) ↓ | | |
| 0.5 | 4283.87 | 7.38 |
| 1.0 | 5837.38 | 7.28 |
| **1.5** | **8642.42** | 9.46 |
| 2.0 | 7624.98 | 3.47 |

S.D. = Standard Deviation (n=3)

**Table 45:** Effect of different concentrations of Lactose and ammonium chloride on production of alkaline protease from *Brevibacterium casei* LAP223

| Isolate → | *Brevibacterium casei* LAP223 | |
| --- | --- | --- |
| Lactose (%) ↓ | Alkaline protease production | |
|  | U/mL | ± S.D. |
| 0.5 | 4539.92 | 7.45 |
| 1.0 | 5833.27 | 7.28 |
| **1.5** | **7642.27** | 8.39 |
| 2.0 | 6452.12 | 5.47 |
| Ammonium chloride (%) ↓ | | |
| 0.5 | 5193.03 | 4.31 |
| 1.0 | 5974.85 | 9.35 |
| **1.5** | **8942.42** | 7.42 |
| 2.0 | 7844.87 | 9.42 |

S.D. = Standard Deviation (n=3)

**Table 46:** Effect of various proteinaceous substrates on catalytic activity of partially purified alkaline protease from *Halomonas venusta* LAP515

| **Substrates**  **(1 %)** | **Enzyme activity (U/mL)** | **± S.D.** | **Relative enzyme activity (%)** |
| --- | --- | --- | --- |
| Casein | **6043** | 4.37 | 100 |
| Hemoglobin | 6040 | 5.23 | 99.95 |
| BSA | 6036 | 2.95 | 99.88 |
| Egg albumin | 6027 | 6.83 | 99.73 |
| Feather meal | 5945 | 5.94 | 98.38 |

S.D. = Standard Deviation (n=3)

**Table 47:** Effect of various proteinaceous substrates on catalytic activity of partially purified alkaline protease from *Brachybacterium* sp. LAP214

| **Substrates**  **(1 %)** | **Enzyme activity (U/mL)** | **± S.D.** | **Relative enzyme activity (%)** |
| --- | --- | --- | --- |
| Casein | **7233.65** | 2.93 | 100.00 |
| BSA | 7204.22 | 5.48 | 99.59 |
| Egg albumin | 7145.32 | 4.39 | 98.78 |
| Feather meal | 7066.28 | 4.22 | 97.69 |
| Hemoglobin | 6973.29 | 3.94 | 96.40 |

S.D. = Standard Deviation (n=3)

**Table 48:** Effect of various proteinaceous substrates on catalytic activity of partially purified alkaline protease from *Bacillus pseudofirmus* LAP220

| **Substrates**  **(1 %)** | **Enzyme activity (U/mL)** | **± S.D.** | **Relative enzyme activity (%)** |
| --- | --- | --- | --- |
| Casein | **6076.87** | 9.98 | 100.00 |
| BSA | 6063.73 | 10.39 | 99.78 |
| Egg albumin | 5986.37 | 7.95 | 98.51 |
| Feather meal | 5902.72 | 7.21 | 97.13 |
| Hemoglobin | 5594.24 | 8.48 | 92.06 |

S.D. = Standard Deviation (n=3)

**Table 49:** Effect of various proteinaceous substrates on catalytic activity of partially purified alkaline protease from *Bacillus cohnii* LAP217

| **Substrates**  **(1 %)** | **Enzyme activity (U/mL)** | **± S.D.** | **Relative enzyme activity (%)** |
| --- | --- | --- | --- |
| Casein | **8067.48** | 7.82 | 100.00 |
| BSA | 7982.91 | 5.38 | 98.95 |
| Egg albumin | 7623.82 | 8.49 | 94.50 |
| Hemoglobin | 7192.81 | 4.29 | 89.16 |
| Feather meal | 6710.29 | 8.44 | 83.18 |

S.D. = Standard Deviation (n=3)

**Table 50:** Effect of various proteinaceous substrates on catalytic activity of partially purified alkaline protease from *Brevibacterium casei* LAP223

| **Substrates**  **(1 %)** | **Enzyme activity (U/mL)** | **± S.D.** | **Relative enzyme activity (%)** |
| --- | --- | --- | --- |
| Casein | 8154.65 | 4.31 | 100.00 |
| BSA | 7883.62 | 5.38 | 96.68 |
| Egg albumin | 6758.33 | 3.92 | 82.88 |
| Feather meal | 6533.93 | 7.28 | 80.13 |
| Hemoglobin | 5373.29 | 3.38 | 65.89 |

S.D. = Standard Deviation (n=3)

**Table 51:** Effect of different concentrations of casein on catalytic activity of alkaline protease from *Halomonas venusta* LAP515

| **Casein (mg/mL)**  **[s]** | **Enzyme activity (U/mL)** | **± S.D.** | **Relative enzyme activity (%)** | **Enzyme activity (U/mg)**  **[v]** | **1/[S]** | **1/[v]** |
| --- | --- | --- | --- | --- | --- | --- |
| 2 | 1703.67 | 8.64 | 28.333 | 170.367 | 0.5000 | 0.00587 |
| 4 | 2801.58 | 9.04 | 46.592 | 280.158 | 0.2500 | 0.00357 |
| 6 | 3846.43 | 10.84 | 63.969 | 384.643 | 0.1667 | 0.00260 |
| 8 | 4911.75 | 11.44 | 81.686 | 491.175 | 0.1250 | 0.00204 |
| **10** | **6012.99** | 14.39 | 100 | 601.299 | 0.1000 | 0.00166 |
| 12 | 4751.18 | 12.28 | 79.015 | 475.118 | 0.0833 | 0.00210 |

**Table 52:** Effect of different concentrations of casein on catalytic activity of alkaline protease from *Brachybacterium* sp. LAP214

| **Casein (mg/mL)**  **[s]** | **Enzyme activity (U/mL)** | **± S.D.** | **Relative enzyme activity (%)** | **Enzyme activity (U/mg)**  **[v]** | **1/[S]** | **1/[v]** |
| --- | --- | --- | --- | --- | --- | --- |
| 2 | 4335.65 | 10.33 | 59.937 | 394.15 | 0.500 | 0.0025 |
| 4 | 5465.65 | 12.54 | 75.559 | 496.8773 | 0.250 | 0.0020 |
| 6 | 6576.65 | 13.22 | 90.917 | 597.8773 | 0.167 | 0.0016 |
| 8 | 7056.87 | 11.65 | 97.556 | 641.5336 | 0.125 | 0.0016 |
| **10** | **7233.65** | 2.93 | 100 | 657.6045 | 0.100 | 0.0015 |
| 12 | 7143.76 | 8.42 | 98.757 | 649.4327 | 0.083 | 0.0015 |

**Table 53:** Effect of different concentrations of casein on catalytic activity of alkaline protease from *Bacillus pseudofirmus* LAP220

| **Casein (mg/mL)**  **[s]** | **Enzyme activity (U/mL)** | **± S.D.** | **Relative enzyme activity (%)** | **Enzyme activity (U/mg)**  **[v]** | **1/[S]** | **1/[v]** |
| --- | --- | --- | --- | --- | --- | --- |
| 2 | 1933.32 | 10.57 | 31.781 | 175.7564 | 0.500 | 0.0057 |
| 4 | 2522.53 | 12.48 | 41.467 | 229.3209 | 0.250 | 0.0044 |
| 6 | 2570.24 | 14.49 | 42.251 | 233.6582 | 0.167 | 0.0043 |
| 8 | 4663.33 | 22.57 | 76.659 | 423.9391 | 0.125 | 0.0024 |
| **10** | **6083.21** | 7.980 | 100 | 553.0191 | 0.100 | 0.0018 |
| 12 | 3853.33 | 24.48 | 63.344 | 350.3027 | 0.083 | 0.0029 |

**Table 54:** Effect of different concentrations of casein on catalytic activity of alkaline protease from *Bacillus cohnii* LAP217

| **Casein (mg/mL)**  **[s]** | **Enzyme activity (U/mL)** | **± S.D.** | **Relative enzyme activity (%)** | **Enzyme activity (U/mg)**  **[v]** | **1/[S]** | **1/[v]** |
| --- | --- | --- | --- | --- | --- | --- |
| 2 | 4032.28 | 12.88 | 49.948 | 403.228 | 0.500 | 0.00248 |
| 4 | 7223.24 | 11.28 | 89.474 | 722.324 | 0.250 | 0.00138 |
| 6 | 7998.22 | 8.94 | 99.074 | 799.822 | 0.167 | 0.00125 |
| 8 | 8012.77 | 13.38 | 99.254 | 801.277 | 0.125 | 0.00125 |
| **10** | **8072.98** | 7.82 | 100 | 807.298 | 0.100 | 0.00124 |
| 12 | 8067.26 | 4.93 | 99.929 | 806.726 | 0.083 | 0.00124 |

**Table 55:** Effect of different concentrations of casein on catalytic activity of alkaline protease from *Brevibacterium casei* LAP223

| **Casein (mg/mL)**  **[s]** | **Enzyme activity (U/mL)** | **± S.D.** | **Relative enzyme activity (%)** | **Enzyme activity (U/mg)**  **[v]** | **1/[S]** | **1/[v]** |
| --- | --- | --- | --- | --- | --- | --- |
| 2 | 2276.28 | 4.83 | 27.633 | 227.628 | 0.500 | 0.004393 |
| 4 | 4763.82 | 12.81 | 57.831 | 476.382 | 0.250 | 0.002099 |
| 6 | 5621.22 | 8.29 | 68.239 | 562.122 | 0.167 | 0.001779 |
| 8 | 7382.28 | 21.73 | 89.618 | 738.228 | 0.125 | 0.001355 |
| **10** | **8237.49** | 5.37 | 100 | 823.749 | 0.100 | 0.001214 |
| 12 | 7042.82 | 11.98 | 85.497 | 704.282 | 0.083 | 0.001420 |

**Table 56:** Effect of pH on catalytic activity and stability of alkaline protease from *Halomonas venusta* LAP515

| pH | Control (without pre-incubation of LAP515 protease at selected pH) | | | | After 15 min of pre-incubation | | |
| --- | --- | --- | --- | --- | --- | --- | --- |
|  | Enzyme activity (**U/mL**) | **± S.D.** | Relative enzyme activity (%) | Initial activity (considered as 100 %) | Enzyme activity (**U/mL**) | **± S.D.** | Residual activity (%) |
| 6 | 4755.24 | 12.54 | 54.312 | 100 | 3802.93 | 12.56 | 79.973 |
| 7 | 5193.87 | 17.92 | 59.321 | 100 | 4184.39 | 11.39 | 80.564 |
| 8 | 5494.38 | 11.43 | 62.754 | 100 | 5093.38 | 13.86 | 92.702 |
| 9 | 5986.45 | 9.67 | 68.374 | 100 | 5586.59 | 15.49 | 93.321 |
| 10 | 6012.99 | 11.85 | 68.677 | 100 | 5687.87 | 7.53 | 94.593 |
| 11 | 7854.49 | 7.58 | 89.710 | 100 | 7484.35 | 9.94 | 95.288 |
| **12** | 8755.46 | 12.86 | 100.00 | 100 | 8445.67 | 10.45 | 96.462 |
| 13 | 6954.39 | 11.34 | 79.429 | 100 | 6245.45 | 11.38 | 89.806 |

**Table 56** (continued):

| pH | After 30 min of pre-incubation | | | After 45 min of pre-incubation | | |
| --- | --- | --- | --- | --- | --- | --- |
|  | Enzyme activity (**U/mL**) | **± S.D.** | Residual activity (%) | Enzyme activity (**U/mL**) | **± S.D.** | Residual activity (%) |
| 6 | 3776.95 | 3.67 | 79.427 | 3574.39 | 12.65 | 75.167 |
| 7 | 4176.67 | 10.85 | 80.415 | 3985.58 | 13.54 | 76.736 |
| 8 | 5002.83 | 9.75 | 91.054 | 4876.95 | 14.37 | 88.763 |
| 9 | 5502.48 | 8.73 | 91.916 | 5486.59 | 11.65 | 91.650 |
| 10 | 5612.45 | 11.84 | 93.339 | 5573.34 | 8.05 | 92.688 |
| 11 | 7376.48 | 12.54 | 93.914 | 7285.49 | 9.94 | 92.756 |
| **12** | 8343.87 | 9.84 | 95.299 | 8256.49 | 4.97 | 94.301 |
| 13 | 6129.38 | 11.48 | 88.137 | 5764.45 | 5.89 | 82.889 |

**Table 56** (continued):

| pH | After 60 min of pre-incubation | | |
| --- | --- | --- | --- |
|  | Enzyme activity (**U/mL**) | **± S.D.** | Residual activity (%) |
| 6 | 3023.39 | 11.39 | 63.580 |
| 7 | 3900.33 | 10.38 | 75.095 |
| 8 | 4763.85 | 13.39 | 86.704 |
| 9 | 5383.76 | 12.39 | 89.932 |
| 10 | 5445.28 | 11.39 | 90.559 |
| 11 | 7184.48 | 12.35 | 91.470 |
| **12** | 8083.49 | 14.49 | 92.325 |
| 13 | 4095.59 | 12.39 | 58.892 |

S.D. = Standard Deviation (n=3)

**Table 57:** Effect of pH on catalytic activity and stability of alkaline protease from *Brachybacterium* sp. LAP214

| pH | Control (without pre-incubation of LAP214 protease at selected pH) | | | | After 15 min of pre-incubation | | |
| --- | --- | --- | --- | --- | --- | --- | --- |
|  | Enzyme activity (**U/mL**) | **± S.D.** | Relative enzyme activity (%) | Initial activity (considered as 100 %) | Enzyme activity (**U/mL**) | **± S.D.** | Residual activity (%) |
| 6 | 4584.39 | 12.95 | 51.070 | 100 | 4083.29 | 3.65 | 89.069 |
| 7 | 4874.39 | 11.43 | 54.301 | 100 | 4365.93 | 4.87 | 89.569 |
| 8 | 5372.29 | 9.45 | 59.847 | 100 | 5043.53 | 3.65 | 93.880 |
| 9 | 6473.39 | 16.94 | 72.113 | 100 | 6095.65 | 5.87 | 94.165 |
| 10 | 7233.65 | 12.54 | 80.583 | 100 | 7114.36 | 9.8 | 98.351 |
| **11** | 8976.68 | 13.6 | 100.00 | 100 | 8845.67 | 11.43 | 98.541 |
| 12 | 8185.46 | 11.64 | 91.186 | 100 | 8043.54 | 15.45 | 98.266 |
| 13 | 7597.93 | 7.56 | 84.641 | 100 | 7293.82 | 9.56 | 95.997 |

**Table 57** (continued):

| pH | After 30 min of pre-incubation | | | After 45 min of pre-incubation | | |
| --- | --- | --- | --- | --- | --- | --- |
|  | Enzyme activity (**U/mL**) | **± S.D.** | Residual activity (%) | Enzyme activity (**U/mL**) | **± S.D.** | Residual activity (%) |
| 6 | 3912.56 | 12.54 | 85.345 | 3193.43 | 10.34 | 69.659 |
| 7 | 4211.34 | 9.67 | 86.397 | 4155.56 | 9.76 | 85.253 |
| 8 | 4804.62 | 5.76 | 89.433 | 4593.45 | 8.56 | 85.503 |
| 9 | 5894.56 | 3.54 | 91.058 | 5783.32 | 6.76 | 89.340 |
| 10 | 7045.36 | 7.69 | 97.397 | 6904.34 | 5.98 | 95.448 |
| **11** | 8745.26 | 11.54 | 97.422 | 8695.56 | 9.45 | 96.868 |
| 12 | 7905.56 | 10.45 | 96.581 | 7464.09 | 4.42 | 91.187 |
| 13 | 7082.5 | 10.35 | 93.216 | 6691.29 | 4.87 | 88.067 |

**Table 57** (continued):

| pH | After 60 min of pre-incubation | | |
| --- | --- | --- | --- |
|  | Enzyme activity (**U/mL**) | **± S.D.** | Residual activity (%) |
| 6 | 3085.59 | 6.87 | 67.306 |
| 7 | 4033.39 | 11.54 | 82.747 |
| 8 | 4484.49 | 10.43 | 83.474 |
| 9 | 5683.35 | 9.34 | 87.796 |
| 10 | 6756.03 | 6.57 | 93.397 |
| **11** | 8433.98 | 5.37 | 93.954 |
| 12 | 7220.34 | 9.47 | 88.209 |
| 13 | 6465.47 | 11.45 | 85.095 |

S.D. = Standard Deviation (n=3)

**Table 58:** Effect of pH on catalytic activity and stability of alkaline protease from *Bacillus pseudofirmus* LAP220

| pH | Control (without pre-incubation of LAP220 protease at selected pH) | | | | After 15 min of pre-incubation | | |
| --- | --- | --- | --- | --- | --- | --- | --- |
|  | Enzyme activity (**U/mL**) | **± S.D.** | Relative enzyme activity (%) | Initial activity (considered as 100 %) | Enzyme activity (**U/mL**) | **± S.D.** | Residual activity (%) |
| 6 | 4800.39 | 2.45 | 60.122 | 100 | 4132.24 | 9.65 | 86.081 |
| 7 | 5274.39 | 4.65 | 66.059 | 100 | 4904.35 | 12.64 | 92.984 |
| 8 | 5584.39 | 4.78 | 69.941 | 100 | 5194.35 | 11.98 | 93.016 |
| 9 | 5984.39 | 5.25 | 74.951 | 100 | 5735.24 | 10.64 | 95.837 |
| 10 | 6083.21 | 4.78 | 76.189 | 100 | 5946.38 | 9.67 | 97.751 |
| 11 | 7129.34 | 7.89 | 89.291 | 100 | 7043.99 | 8.45 | 98.803 |
| **12** | 7984.38 | 5.67 | 100.00 | 100 | 7900.32 | 11.46 | 98.947 |
| 13 | 6832.28 | 7.64 | 85.571 | 100 | 6702.94 | 17.67 | 98.107 |

**Table 58** (continued):

| pH | After 30 min of pre-incubation | | | After 45 min of pre-incubation | | |
| --- | --- | --- | --- | --- | --- | --- |
|  | Enzyme activity (**U/mL**) | **± S.D.** | Residual activity (%) | Enzyme activity (**U/mL**) | **± S.D.** | Residual activity (%) |
| 6 | 4032.43 | 4.65 | 84.002 | 3993.34 | 5.74 | 83.188 |
| 7 | 4535.45 | 7.67 | 85.990 | 4454.39 | 3.87 | 84.453 |
| 8 | 4942.45 | 4.2 | 88.505 | 4883.45 | 5.85 | 87.448 |
| 9 | 5493.42 | 4.51 | 91.796 | 5348.24 | 6.43 | 89.370 |
| 10 | 5745.24 | 2.67 | 94.444 | 5493.45 | 9.26 | 90.305 |
| 11 | 6843.22 | 4.98 | 95.987 | 6694.35 | 3.98 | 93.899 |
| **12** | 7845.35 | 3.58 | 98.259 | 7693.43 | 9.65 | 96.356 |
| 13 | 6234.35 | 9.44 | 91.248 | 6193.32 | 10.54 | 90.648 |

**Table 58** (continued):

| pH | After 60 min of pre-incubation | | |
| --- | --- | --- | --- |
|  | Enzyme activity (**U/mL**) | **± S.D.** | Residual activity (%) |
| 6 | 3843.19 | 2.56 | 80.060 |
| 7 | 4343.53 | 4.35 | 82.351 |
| 8 | 4692.43 | 7.98 | 84.028 |
| 9 | 5165.48 | 9.65 | 86.316 |
| 10 | 5255.32 | 8.53 | 86.391 |
| 11 | 6553.47 | 3.47 | 91.923 |
| **12** | 7457.49 | 8.65 | 93.401 |
| 13 | 5852.28 | 8.43 | 85.656 |

S.D. = Standard Deviation (n=3)

**Table 59:** Effect of pH on catalytic activity and stability of alkaline protease from *Bacillus cohnii* LAP217

| pH | Control (without pre-incubation of LAP217 protease at selected pH) | | | | After 15 min of pre-incubation | | |
| --- | --- | --- | --- | --- | --- | --- | --- |
|  | Enzyme activity (**U/mL**) | **± S.D.** | Relative enzyme activity (%) | Initial activity (considered as 100 %) | Enzyme activity (**U/mL**) | **± S.D.** | Residual activity (%) |
| 6 | 4623.17 | 3.84 | 57.267 | 100 | 3894.49 | 2.65 | 84.239 |
| 7 | 4731.39 | 4.38 | 58.608 | 100 | 4183.29 | 4.36 | 88.416 |
| 8 | 5242.31 | 5.63 | 64.936 | 100 | 4585.59 | 5.31 | 87.473 |
| 9 | 6773.47 | 7.42 | 83.903 | 100 | 5973.21 | 4.87 | 88.185 |
| **10** | 8072.98 | 8.76 | 100.00 | 100 | 7803.74 | 9.65 | 96.665 |
| 11 | 7453.32 | 9.53 | 92.324 | 100 | 7123.43 | 7.43 | 95.574 |
| 12 | 6328.28 | 11.45 | 78.388 | 100 | 5993.04 | 8.23 | 94.703 |
| 13 | 5748.28 | 12.65 | 71.204 | 100 | 5183.32 | 10.43 | 90.172 |

**Table 59** (continued):

| pH | After 30 min of pre-incubation | | | After 45 min of pre-incubation | | |
| --- | --- | --- | --- | --- | --- | --- |
|  | Enzyme activity (**U/mL**) | **± S.D.** | Residual activity (%) | Enzyme activity (**U/mL**) | **± S.D.** | Residual activity (%) |
| 6 | 3704.32 | 4.65 | 80.125 | 3654.76 | 3.65 | 79.053 |
| 7 | 3982.14 | 7.4 | 84.164 | 3896.43 | 4.73 | 82.353 |
| 8 | 4442.83 | 3.98 | 84.749 | 4383.29 | 4.29 | 83.614 |
| 9 | 5883.29 | 8.05 | 86.858 | 5776.02 | 5.16 | 85.274 |
| **10** | 7749.93 | 5.36 | 95.998 | 7643.29 | 6.32 | 94.677 |
| 11 | 7054.29 | 7.16 | 94.646 | 6944.29 | 7.54 | 93.170 |
| 12 | 5893.29 | 11.54 | 93.126 | 5638.28 | 8.45 | 89.097 |
| 13 | 5032.23 | 14.32 | 87.543 | 4938.29 | 9.53 | 85.909 |

**Table 59** (continued):

| pH | After 60 min of pre-incubation | | |
| --- | --- | --- | --- |
|  | Enzyme activity (**U/mL**) | **± S.D.** | Residual activity (%) |
| 6 | 3183.32 | 12.54 | 68.856 |
| 7 | 3584.28 | 9.54 | 75.755 |
| 8 | 4283.29 | 7.26 | 81.706 |
| 9 | 5632.27 | 7.54 | 83.152 |
| **10** | 7034.23 | 9.65 | 87.133 |
| 11 | 5182.27 | 5.36 | 69.530 |
| 12 | 4134.28 | 4.26 | 65.330 |
| 13 | 3723.28 | 4.11 | 64.772 |

S.D. = Standard Deviation (n=3)

**Table 60:** Effect of pH on catalytic activity and stability of alkaline protease from *Brevibacterium casei* LAP223

| pH | Control (without pre-incubation of LAP223 protease at selected pH) | | | | After 15 min of pre-incubation | | |
| --- | --- | --- | --- | --- | --- | --- | --- |
|  | Enzyme activity (**U/mL**) | **± S.D.** | Relative enzyme activity (%) | Initial activity (considered as 100 %) | Enzyme activity (**U/mL**) | **± S.D.** | Residual activity (%) |
| 6 | 4853.28 | 4.53 | 50.856 | 100 | 4533.28 | 7.49 | 93.407 |
| 7 | 5934.13 | 6.33 | 62.182 | 100 | 5654.35 | 4.25 | 95.285 |
| 8 | 6284.28 | 8.95 | 65.851 | 100 | 6084.34 | 8.52 | 96.818 |
| 9 | 7343.22 | 10.03 | 76.947 | 100 | 7145.35 | 10.34 | 97.305 |
| 10 | 8237.49 | 2.42 | 86.318 | 100 | 8054.35 | 11.36 | 97.777 |
| **11** | 9543.24 | 11.04 | 100.00 | 100 | 9423.45 | 9.32 | 98.745 |
| 12 | 8134.29 | 10.43 | 85.236 | 100 | 7670.25 | 7.42 | 94.295 |
| 13 | 6933.58 | 9.43 | 72.654 | 100 | 6082.30 | 6.35 | 87.722 |

**Table 60** (continued):

| pH | After 30 min of pre-incubation | | | After 45 min of pre-incubation | | |
| --- | --- | --- | --- | --- | --- | --- |
|  | Enzyme activity (**U/mL**) | **± S.D.** | Residual activity (%) | Enzyme activity (**U/mL**) | **± S.D.** | Residual activity (%) |
| 6 | 4483.39 | 3.56 | 92.379 | 4031.52 | 3.28 | 83.068 |
| 7 | 5295.94 | 5.76 | 89.245 | 5193.42 | 5.48 | 87.518 |
| 8 | 5633.24 | 7.86 | 89.640 | 5563.42 | 5.02 | 88.529 |
| 9 | 6794.42 | 9.76 | 92.526 | 6543.22 | 4.96 | 89.106 |
| 10 | 7853.32 | 10.65 | 95.336 | 7635.09 | 6.93 | 92.687 |
| **11** | 9153.42 | 11.64 | 95.915 | 9040.02 | 7.05 | 94.727 |
| 12 | 7656.42 | 9.64 | 94.125 | 7194.06 | 4.66 | 88.441 |
| 13 | 6043.32 | 9.66 | 87.160 | 5893.38 | 7.35 | 84.998 |

**Table 60** (continued):

| pH | After 60 min of pre-incubation | | |
| --- | --- | --- | --- |
|  | Enzyme activity (**U/mL**) | **± S.D.** | Residual activity (%) |
| 6 | 3984.52 | 5.36 | 82.100 |
| 7 | 5053.42 | 8.65 | 85.159 |
| 8 | 5435.31 | 9.43 | 86.491 |
| 9 | 6443.35 | 3.27 | 87.746 |
| 10 | 7453.32 | 2.67 | 90.480 |
| **11** | 8764.88 | 8.67 | 91.844 |
| 12 | 6988.54 | 9.45 | 85.915 |
| 13 | 4884.89 | 11.22 | 70.453 |

S.D. = Standard Deviation (n=3)

**Table 61:** Effect of temperature on catalytic activity and stability of alkaline protease from *Halomonas venusta* LAP515

| Temperature | Control (without pre-incubation of LAP515 protease at selected temperatures) | | | | After 15 min of pre-incubation | | |
| --- | --- | --- | --- | --- | --- | --- | --- |
|  | Enzyme activity (**U/mL**) | **± S.D.** | Relative enzyme activity (%) | Initial activity (considered as 100 %) | Enzyme activity (**U/mL**) | **± S.D.** | Residual activity (%) |
| 20 ⁰C | 5293.53 | 4.67 | 84.239 | 100 | 5091.46 | 2.86 | 96.183 |
| 30 ⁰C | 6012.99 | 11.85 | 95.688 | 100 | 5812.24 | 3.58 | 96.661 |
| **40 ⁰C** | 6283.96 | 6.87 | 100.00 | 100 | 6151.28 | 7.47 | 97.889 |
| 50 ⁰C | 5583.56 | 12.75 | 88.854 | 100 | 5196.35 | 8.26 | 93.065 |
| 60 ⁰C | 4832.76 | 12.36 | 76.906 | 100 | 4446.21 | 9.52 | 92.001 |
| 70 ⁰C | 4102.38 | 9.54 | 65.283 | 100 | 3752.59 | 5.73 | 91.473 |

**Table 61** (continued):

| Temperature | After 30 min of pre-incubation | | | After 45 min of pre-incubation | | |
| --- | --- | --- | --- | --- | --- | --- |
|  | Enzyme activity (**U/mL**) | **± S.D.** | Residual activity (%) | Enzyme activity (**U/mL**) | **± S.D.** | Residual activity (%) |
| 20 ⁰C | 5086.45 | 4.37 | 96.088 | 5037.43 | 3.67 | 95.162 |
| 30 ⁰C | 5800.25 | 4.75 | 96.462 | 5733.25 | 4.72 | 95.348 |
| **40 ⁰C** | 6106.80 | 6.36 | 97.181 | 5995.29 | 7.59 | 95.406 |
| 50 ⁰C | 5395.22 | 7.27 | 96.627 | 5105.37 | 9.86 | 91.436 |
| 60 ⁰C | 4509.79 | 4.91 | 93.317 | 4412.48 | 8.47 | 91.304 |
| 70 ⁰C | 3507.43 | 5.99 | 85.497 | 3146.34 | 9.21 | 76.695 |

**Table 61** (continued):

| Temperature | After 60 min of pre-incubation | | |
| --- | --- | --- | --- |
|  | Enzyme activity (**U/mL**) | **± S.D.** | Residual activity (%) |
| 20 ⁰C | 5032.43 | 3.67 | 95.068 |
| 30 ⁰C | 5720.36 | 6.36 | 95.133 |
| **40 ⁰C** | 5992.36 | 4.21 | 95.360 |
| 50 ⁰C | 4053.39 | 3.75 | 72.595 |
| 60 ⁰C | 3303.88 | 2.73 | 68.364 |
| 70 ⁰C | 2694.58 | 4.11 | 65.683 |

S.D. = Standard Deviation (n=3)

**Table 62:** Effect of temperature on catalytic activity and stability of alkaline protease from *Brachybacterium* sp. LAP214

| Temperature | Control  (without pre-incubation of LAP214 protease at selected temperatures) | | | | After 15 min of pre-incubation | | |
| --- | --- | --- | --- | --- | --- | --- | --- |
|  | Enzyme activity (**U/mL**) | **± S.D.** | Relative enzyme activity (%) | Initial activity (considered as 100 %) | Enzyme activity (**U/mL**) | **± S.D.** | Residual activity (%) |
| 20 ⁰C | 7192.39 | 2.43 | 99.430 | 100 | 7192.39 | 2.43 | 100.000 |
| **30 ⁰C** | 7233.65 | 12.54 | 100.00 | 100 | 7233.28 | 4.85 | 99.995 |
| 40 ⁰C | 6123.27 | 4.39 | 84.650 | 100 | 6121.43 | 5.43 | 99.970 |
| 50 ⁰C | 6063.31 | 9.43 | 83.821 | 100 | 6004.32 | 8.42 | 99.027 |
| 60 ⁰C | 5942.39 | 4.38 | 82.149 | 100 | 5843.24 | 8.54 | 98.331 |
| 70 ⁰C | 5291.37 | 3.92 | 73.149 | 100 | 5183.74 | 9.21 | 97.966 |

**Table 62** (continued):

| Temperature | After 30 min of pre-incubation | | | After 45 min of pre-incubation | | |
| --- | --- | --- | --- | --- | --- | --- |
|  | Enzyme activity (**U/mL**) | **± S.D.** | Residual activity (%) | Enzyme activity (**U/mL**) | **± S.D.** | Residual activity (%) |
| 20 ⁰C | 7180.43 | 4.95 | 99.834 | 7102.35 | 4.65 | 98.748 |
| **30 ⁰C** | 7221.74 | 3.85 | 99.835 | 7213.36 | 7.25 | 99.725 |
| 40 ⁰C | 6032.84 | 4.23 | 98.523 | 6011.35 | 7.45 | 98.202 |
| 50 ⁰C | 5943.82 | 8.95 | 98.029 | 5742.47 | 9.12 | 95.639 |
| 60 ⁰C | 5752.35 | 7.54 | 96.802 | 5145.63 | 6.29 | 88.061 |
| 70 ⁰C | 5074.98 | 9.32 | 95.911 | 4467.25 | 7.14 | 86.178 |

**Table 62** (continued):

| Temperature | After 60 min of pre-incubation | | |
| --- | --- | --- | --- |
|  | Enzyme activity (**U/mL**) | **± S.D.** | Residual activity (%) |
| 20 ⁰C | 7165.00 | 4.64 | 99.619 |
| **30 ⁰C** | 7207.54 | 7.35 | 99.639 |
| 40 ⁰C | 5954.63 | 4.62 | 97.246 |
| 50 ⁰C | 5143.65 | 9.65 | 84.832 |
| 60 ⁰C | 4956.24 | 6.35 | 83.405 |
| 70 ⁰C | 4194.35 | 7.24 | 79.268 |

S.D. = Standard Deviation (n=3)

**Table 63:** Effect of temperature on catalytic activity and stability of alkaline protease from *Bacillus pseudofirmus* LAP220

| Temperature | Control (without pre-incubation of LAP220 protease at selected temperatures) | | | | After 15 min of pre-incubation | | |
| --- | --- | --- | --- | --- | --- | --- | --- |
|  | Enzyme activity (**U/mL**) | **± S.D.** | Relative enzyme activity (%) | Initial activity (considered as 100 %) | Enzyme activity (**U/mL**) | **± S.D.** | Residual activity (%) |
| 20 ⁰C | 6011.63 | 5.76 | 71.114 | 100 | 5992.67 | 2.54 | 99.685 |
| 30 ⁰C | 6083.21 | 8.64 | 71.961 | 100 | 6070.43 | 6.51 | 99.790 |
| 40 ⁰C | 7439.39 | 9.64 | 88.003 | 100 | 7429.45 | 2.37 | 99.866 |
| **50 ⁰C** | 8453.53 | 8.23 | 100.00 | 100 | 8443.25 | 4.75 | 99.878 |
| 60 ⁰C | 8133.56 | 9.74 | 96.215 | 100 | 8021.36 | 6.50 | 98.621 |
| 70 ⁰C | 7085.34 | 11.75 | 83.815 | 100 | 6987.58 | 6.32 | 98.620 |

**Table 63** (continued):

| Temperature | After 30 min of pre-incubation | | | After 45 min of pre-incubation | | |
| --- | --- | --- | --- | --- | --- | --- |
|  | Enzyme activity (**U/mL**) | **± S.D.** | Residual activity (%) | Enzyme activity (**U/mL**) | **± S.D.** | Residual activity (%) |
| 20 ⁰C | 5990.54 | 4.65 | 99.649 | 5983.24 | 3.84 | 99.528 |
| 30 ⁰C | 6051.46 | 7.35 | 99.478 | 6045.32 | 5.42 | 99.377 |
| 40 ⁰C | 7245.90 | 8.37 | 97.399 | 7240.42 | 9.54 | 97.325 |
| **50 ⁰C** | 8431.43 | 2.13 | 99.739 | 8423.4 | 10.42 | 99.644 |
| 60 ⁰C | 8003.54 | 5.88 | 98.401 | 7884.53 | 9.53 | 96.938 |
| 70 ⁰C | 6861.43 | 5.21 | 96.840 | 6092.43 | 9.54 | 85.986 |

**Table 63** (continued):

| Temperature | After 60 min of pre-incubation | | |
| --- | --- | --- | --- |
|  | Enzyme activity (**U/mL**) | **± S.D.** | Residual activity (%) |
| 20 ⁰C | 5974.34 | 4.54 | 99.380 |
| 30 ⁰C | 6012.54 | 5.31 | 98.838 |
| 40 ⁰C | 7132.65 | 9.54 | 95.877 |
| **50 ⁰C** | 8332.65 | 3.56 | 98.570 |
| 60 ⁰C | 6053.00 | 3.90 | 74.420 |
| 70 ⁰C | 5243.32 | 5.41 | 74.002 |

S.D. = Standard Deviation (n=3)

**Table 64:** Effect of temperature on catalytic activity and stability of alkaline protease from *Bacillus cohnii* LAP217

| Temperature | Control (without pre-incubation of LAP217 protease at selected temperatures) | | | | After 15 min of pre-incubation | | |
| --- | --- | --- | --- | --- | --- | --- | --- |
|  | Enzyme activity (**U/mL**) | **± S.D.** | Relative enzyme activity (%) | Initial activity (considered as 100 %) | Enzyme activity (**U/mL**) | **± S.D.** | Residual activity (%) |
| 20 ⁰C | 8071.46 | 4.75 | 87.291 | 100 | 8071.46 | 4.23 | 100.00 |
| 30 ⁰C | 8072.98 | 8.76 | 87.307 | 100 | 8072.37 | 5.47 | 99.992 |
| 40 ⁰C | 8154.76 | 6.46 | 88.192 | 100 | 8152.34 | 5.42 | 99.970 |
| 50 ⁰C | 8794.53 | 6.26 | 95.111 | 100 | 8702.76 | 7.65 | 98.957 |
| **60 ⁰C** | 9246.64 | 8.54 | 100.00 | 100 | 9213.74 | 9.25 | 99.644 |
| 70 ⁰C | 8576.36 | 9.05 | 92.751 | 100 | 8473.25 | 10.54 | 98.798 |

**Table 64** (continued):

| Temperature | After 30 min of pre-incubation | | | After 45 min of pre-incubation | | |
| --- | --- | --- | --- | --- | --- | --- |
|  | Enzyme activity (**U/mL**) | **± S.D.** | Residual activity (%) | Enzyme activity (**U/mL**) | **± S.D.** | Residual activity (%) |
| 20 ⁰C | 8068.34 | 4.52 | 99.961 | 8011.45 | 5.43 | 99.257 |
| 30 ⁰C | 8070.29 | 6.53 | 99.967 | 8029.64 | 9.53 | 99.463 |
| 40 ⁰C | 8103.54 | 7.26 | 99.372 | 7954.39 | 5.12 | 97.543 |
| 50 ⁰C | 8623.53 | 9.43 | 98.056 | 8583.29 | 4.35 | 97.598 |
| **60 ⁰C** | 9154.35 | 3.91 | 99.002 | 9003.43 | 6.53 | 97.370 |
| 70 ⁰C | 8054.34 | 8.64 | 93.913 | 7974.88 | 2.46 | 92.987 |

**Table 64** (continued):

| Temperature | After 60 min of pre-incubation | | |
| --- | --- | --- | --- |
|  | Enzyme activity (**U/mL**) | **± S.D.** | Residual activity (%) |
| 20 ⁰C | 7912.45 | 4.83 | 98.030 |
| 30 ⁰C | 7912.52 | 5.26 | 98.012 |
| 40 ⁰C | 7853.48 | 7.45 | 96.305 |
| 50 ⁰C | 8482.45 | 9.42 | 96.451 |
| **60 ⁰C** | 8745.25 | 9.23 | 94.578 |
| 70 ⁰C | 7253.95 | 7.43 | 84.581 |

S.D. = Standard Deviation (n=3)

**Table 65:** Effect of temperature on catalytic activity and stability of alkaline protease from *Brevibacterium casei* LAP223

| Temperature | Control (without pre-incubation of LAP223 protease at selected temperatures) | | | | After 15 min of pre-incubation | | |
| --- | --- | --- | --- | --- | --- | --- | --- |
|  | Enzyme activity (**U/mL**) | **± S.D.** | Relative enzyme activity (%) | Initial activity (considered as 100 %) | Enzyme activity (**U/mL**) | **± S.D.** | Residual activity (%) |
| 20 ⁰C | 8074.34 | 2.43 | 80.712 | 100 | 8072.14 | 3.84 | 99.973 |
| 30 ⁰C | 8237.49 | 2.42 | 82.343 | 100 | 8211.34 | 5.28 | 99.683 |
| 40 ⁰C | 8943.29 | 4.24 | 89.398 | 100 | 8920.19 | 9.41 | 99.742 |
| **50 ⁰C** | 10003.94 | 5.25 | 100.00 | 100 | 9991.34 | 2.48 | 99.874 |
| 60 ⁰C | 9743.25 | 9.42 | 97.395 | 100 | 9722.38 | 5.48 | 99.786 |
| 70 ⁰C | 9183.49 | 5.14 | 91.799 | 100 | 9129.43 | 4.29 | 99.411 |

**Table 65** (continued):

| Temperature | After 30 min of pre-incubation | | | After 45 min of pre-incubation | | |
| --- | --- | --- | --- | --- | --- | --- |
|  | Enzyme activity (**U/mL**) | **± S.D.** | Residual activity (%) | Enzyme activity (**U/mL**) | **± S.D.** | Residual activity (%) |
| 20 ⁰C | 8063.24 | 3.42 | 99.863 | 8059.34 | 4.39 | 99.814 |
| 30 ⁰C | 8204.28 | 5.93 | 99.597 | 8196.37 | 3.82 | 99.501 |
| 40 ⁰C | 8911.32 | 8.12 | 99.643 | 8874.08 | 9.57 | 99.226 |
| **50 ⁰C** | 9983.58 | 9.43 | 99.796 | 9932.38 | 11.38 | 99.285 |
| 60 ⁰C | 9720.34 | 7.43 | 99.765 | 9232.39 | 2.48 | 94.757 |
| 70 ⁰C | 9113.24 | 7.21 | 99.235 | 7392.28 | 8.09 | 80.495 |

**Table 65** (continued):

| Temperature | After 60 min of pre-incubation | | |
| --- | --- | --- | --- |
|  | Enzyme activity (**U/mL**) | **± S.D.** | Residual activity (%) |
| 20 ⁰C | 8052.27 | 4.39 | 99.727 |
| 30 ⁰C | 8112.88 | 3.17 | 98.487 |
| 40 ⁰C | 8809.38 | 9.42 | 98.503 |
| **50 ⁰C** | 9232.74 | 8.41 | 92.291 |
| 60 ⁰C | 8732.70 | 8.43 | 89.628 |
| 70 ⁰C | 6309.92 | 9.63 | 68.709 |

S.D. = Standard Deviation (n=3)

**Table 66:** Effect of various protease inhibitors, surfactants, chelator and oxidizing agent on catalytic efficiency of partially purified alkaline protease from *Halomonas venusta* LAP515

| **Chemicals** | **Concentration** | **Enzyme activity (U/mL)** | **± S.D.** | **Residual activity (%)** |
| --- | --- | --- | --- | --- |
| Control | - | 3142.58 |  | 100 |
| PMSF ^a^ | 1 mM | 1153.91 | 3.65 | 36.719 |
|  | 10 mM | 0 | 0 | 0 |
| DTT ^a^ | 1 mM | 545.39 | 6..94 | 17.355 |
|  | 10 mM | 12.94 | 0.03 | 0.412 |
| Tween 20 ^b^ | 1 % | 3056.95 | 4.62 | 97.275 |
|  | 5 % | 3008.64 | 3.44 | 95.738 |
| Tween 80 ^b^ | 1 % | 3142.53 | 1.17 | 99.998 |
|  | 5 % | 3134.63 | 2.59 | 99.747 |
| Triton X-100 ^b^ | 1 % | 3086.3 | 1.63 | 98.209 |
|  | 5 % | 2854.98 | 2.36 | 90.848 |
| EDTA ^c^ | 1 mM | 3142.57 | 1.47 | 100.000 |
|  | 10 mM | 3135.76 | 0.13 | 99.783 |
|  | 20 mM | 3130.53 | 0.52 | 99.617 |
| H_2_O_2_ ^d^ | 1 % | 3111.45 | 0.54 | 99.009 |
|  | 10 % | 3056.08 | 3.21 | 97.247 |

Abbreviations: S.D. = Standard Deviation (n=3), a-Inhibitor, b-Surfactant, c-Chelator, d-Oxidizing agent.

**Table 67:** Effect of various protease inhibitors, surfactants, chelator and oxidizing agent on catalytic efficiency of partially purified alkaline protease from *Brachybacterium* sp. LAP214

| **Chemicals** | **Concentration** | **Enzyme activity (U/mL)** | **± S.D.** | **Residual activity (%)** |
| --- | --- | --- | --- | --- |
| Control | - | 3110.24 |  | 100 |
| PMSF ^a^ | 1 mM | 2322.41 | 4.29 | 74.670 |
|  | 10 mM | 0 | 0 | 0 |
| DTT ^a^ | 1 mM | 583.31 | 3.53 | 18.755 |
|  | 10 mM | 10.35 | 0.03 | 0.333 |
| Tween 20 ^b^ | 1 % | 3140.37 | 4.35 | 100.969 |
|  | 5 % | 3156.38 | 2.21 | 101.483 |
| Tween 80 ^b^ | 1 % | 3190.36 | 0.03 | 102.576 |
|  | 5 % | 3129.31 | 0.04 | 100.613 |
| Triton X-100 ^b^ | 1 % | 3107.26 | 0.01 | 99.904 |
|  | 5 % | 3017.28 | 1.31 | 97.011 |
| EDTA ^c^ | 1 mM | 3101.27 | 0.01 | 99.712 |
|  | 10 mM | 3098.21 | 0.01 | 99.613 |
|  | 20 mM | 1381.27 | 4.28 | 44.410 |
| H_2_O_2_ ^d^ | 1 % | 3038.29 | 2.18 | 97.687 |
|  | 10 % | 2977.09 | 1.31 | 95.719 |

Abbreviations: S.D. = Standard Deviation (n=3), a-Inhibitor, b-Surfactant, c-Chelator, d-Oxidizing agent.

**Table 68:** Effect of various protease inhibitors, surfactants, chelator and oxidizing agent on catalytic efficiency of partially purified alkaline protease from *Bacillus pseudofirmus* LAP220

| **Chemicals** | **Concentration** | **Enzyme activity (U/mL)** | **± S.D.** | **Residual activity (%)** |
| --- | --- | --- | --- | --- |
| Control | - | 4221.73 |  | 100 |
| PMSF ^a^ | 1 mM | 2933.75 | 4.65 | 69.492 |
|  | 10 mM | 0 | 0 | 0 |
| DTT ^a^ | 1 mM | 4037.81 | 6.57 | 95.643 |
|  | 10 mM | 102.35 | 1.94 | 2.424 |
| Tween 20 ^b^ | 1 % | 3842.48 | 6.78 | 91.017 |
|  | 5 % | 2309.03 | 8.42 | 54.694 |
| Tween 80 ^b^ | 1 % | 4027.25 | 9.66 | 95.393 |
|  | 5 % | 3998.42 | 6.57 | 94.710 |
| Triton X-100 ^b^ | 1 % | 4032.27 | 3.45 | 95.512 |
|  | 5 % | 3944.21 | 7.57 | 93.426 |
| EDTA ^c^ | 1 mM | 4083.27 | 2.46 | 96.720 |
|  | 10 mM | 2932.66 | 2.47 | 69.466 |
|  | 20 mM | 2184.42 | 7.84 | 51.742 |
| H_2_O_2_ ^d^ | 1 % | 3094.29 | 9.64 | 73.294 |
|  | 10 % | 2983.37 | 4.09 | 70.667 |

Abbreviations: S.D. = Standard Deviation (n=3), a-Inhibitor, b-Surfactant, c-Chelator, d-Oxidizing agent.

**Table 69:** Effect of various protease inhibitors, surfactants, chelator and oxidizing agent on catalytic efficiency of partially purified alkaline protease from *Bacillus cohnii* LAP217

| **Chemicals** | **Concentration** | **Enzyme activity (U/mL)** | **± S.D.** | **Residual activity (%)** |
| --- | --- | --- | --- | --- |
| Control | - | 4036.62 |  | 100 |
| PMSF ^a^ | 1 mM | 273.273 | 2.36 | 6.770 |
|  | 10 mM | 0 | 0 | 0 |
| DTT ^a^ | 1 mM | 1932.38 | 11.34 | 47.871 |
|  | 10 mM | 0 | 0 | 0.000 |
| Tween 20 ^b^ | 1 % | 5643.28 | 9.34 | 139.802 |
|  | 5 % | 4016.22 | 0.01 | 99.495 |
| Tween 80 ^b^ | 1 % | 4004.28 | 0.02 | 99.199 |
|  | 5 % | 3822.43 | 2.74 | 94.694 |
| Triton X-100 ^b^ | 1 % | 5172.83 | 6.62 | 128.148 |
|  | 5 % | 3782.22 | 3.47 | 93.698 |
| EDTA ^c^ | 1 mM | 4031.34 | 0.02 | 99.869 |
|  | 10 mM | 4001.34 | 0.01 | 99.126 |
|  | 20 mM | 3974.32 | 0.02 | 98.457 |
| H_2_O_2_ ^d^ | 1 % | 4011.37 | 0.03 | 99.374 |
|  | 10 % | 3084.66 | 0.02 | 76.417 |

Abbreviations: S.D. = Standard Deviation (n=3), a-Inhibitor, b-Surfactant, c-Chelator, d-Oxidizing agent.

**Table 70:** Effect of various protease inhibitors, surfactants, chelator and oxidizing agent on catalytic efficiency of partially purified alkaline protease from *Brevibacterium casei* LAP223

| **Chemicals** | **Concentration** | **Enzyme activity (U/mL)** | **± S.D.** | **Residual activity (%)** |
| --- | --- | --- | --- | --- |
| Control | - | 4100.38 |  | 100.000 |
| PMSF ^a^ | 1 mM | 3943.9 | 2.32 | 96.184 |
|  | 10 mM | 0 | 0 | 0.000 |
| DTT ^a^ | 1 mM | 2834.88 | 4.32 | 69.137 |
|  | 10 mM | 0 | 0 | 0.000 |
| Tween 20 ^b^ | 1 % | 3773.28 | 2.99 | 92.023 |
|  | 5 % | 4002.36 | 1.45 | 97.609 |
| Tween 80 ^b^ | 1 % | 4023.28 | 0.54 | 98.120 |
|  | 5 % | 2823.37 | 4.35 | 68.856 |
| Triton X-100 ^b^ | 1 % | 2837.09 | 8.42 | 69.191 |
|  | 5 % | 4092.34 | 0.12 | 99.804 |
| EDTA ^c^ | 1 mM | 3182.35 | 4.66 | 77.611 |
|  | 10 mM | 3533.75 | 2.41 | 86.181 |
|  | 20 mM | 3288.54 | 5.44 | 80.201 |
| H_2_O_2_ ^d^ | 1 % | 3185.64 | 8.43 | 77.691 |
|  | 10 % | 2967.33 | 2.33 | 72.367 |

Abbreviations: S.D. = Standard Deviation (n=3), a-Inhibitor, b-Surfactant, c-Chelator, d-Oxidizing agent.

**Table 71:** Effect of various metal ions on catalytic efficiency of partially purified alkaline protease from *Halomonas venusta* LAP515

| **Metal ions** | **Concentration**  **(mM)** | **Enzyme activity (U/mL)** | **± S.D.** | **Residual activity (%)** |
| --- | --- | --- | --- | --- |
| Control | - | 3006.32 |  | 100 |
| Mg^2+^ | 1 | 3006.32 | 0.02 | 100 |
|  | 10 | 3183.18 | 0.04 | 105.883 |
|  | 100 | 3015.43 | 0.04 | 100.303 |
| Mn^2+^ | 1 | 3293.28 | 0.34 | 109.545 |
|  | 10 | 3584.11 | 3.28 | 119.219 |
|  | 100 | 3173.32 | 1.92 | 105.555 |
| Cu^2+^ | 1 | 3006.4 | 0.03 | 100.003 |
|  | 10 | 3087.34 | 0.13 | 102.695 |
|  | 100 | 3023.17 | 0.04 | 100.560 |
| Fe^3+^ | 1 | 3006.11 | 0.01 | 99.993 |
|  | 10 | 3783.32 | 1.43 | 125.846 |
|  | 100 | 3537.28 | 2.54 | 117.661 |
| Zn^2+^ | 1 | 3283.27 | 1.33 | 109.212 |
|  | 10 | 2893.27 | 0.35 | 96.240 |
|  | 100 | 1823.23 | 3.25 | 60.647 |
| Ba^2+^ | 1 | 3173.37 | 2.53 | 105.557 |
|  | 10 | 3982.43 | 5.43 | 132.469 |
|  | 100 | 2873.16 | 8.53 | 95.571 |
| Hg^2+^ | 1 | 3006.16 | 0.04 | 99.995 |
|  | 10 | 2873.27 | 3.25 | 95.574 |
|  | 100 | 1002.32 | 6.54 | 33.340 |
| Ca^2+^ | 1 | 3483.27 | 5.46 | 115.865 |
|  | 10 | 4732.84 | 2.21 | 157.430 |
|  | 100 | 3277.39 | 1.58 | 109.017 |
| Na^+^ | 1 | 3004.37 | 0.24 | 99.935 |
|  | 10 | 2998.37 | 0.02 | 99.736 |
|  | 100 | 2763.28 | 3.84 | 91.916 |

Abbreviations: S.D. = Standard Deviation (n=3)

**Table 72:** Effect of various metal ions on catalytic efficiency of partially purified alkaline protease from *Brachybacterium* sp. LAP214

| **Metal ions** | **Concentration**  **(mM)** | **Enzyme activity (U/mL)** | **± S.D.** | **Residual activity (%)** |
| --- | --- | --- | --- | --- |
| Control | - | 3116.48 |  | 100.000 |
| Mg^2+^ | 1 | 3118.84 | 0.24 | 100.076 |
|  | 10 | 4839.28 | 3.47 | 155.280 |
|  | 100 | 4548.11 | 5.47 | 145.937 |
| Mn^2+^ | 1 | 3116.83 | 0.03 | 100.011 |
|  | 10 | 2987.92 | 0.65 | 95.875 |
|  | 100 | 2183.28 | 3.52 | 70.056 |
| Cu^2+^ | 1 | 3111.38 | 0.053 | 99.836 |
|  | 10 | 3982.28 | 7.58 | 127.781 |
|  | 100 | 1733.29 | 7.59 | 55.617 |
| Fe^3+^ | 1 | 3117.28 | 0.02 | 100.026 |
|  | 10 | 2893.28 | 4.75 | 92.838 |
|  | 100 | 1833.28 | 7.53 | 58.825 |
| Zn^2+^ | 1 | 2839.29 | 3.34 | 91.106 |
|  | 10 | 2183.77 | 2.53 | 70.072 |
|  | 100 | 1733.27 | 5.36 | 55.616 |
| Ba^2+^ | 1 | 3115.33 | 0.02 | 99.963 |
|  | 10 | 4740.92 | 2.56 | 152.124 |
|  | 100 | 283.34 | 1.45 | 9.092 |
| Hg^2+^ | 1 | 3111.29 | 3.65 | 99.833 |
|  | 10 | 3102.13 | 1.06 | 99.535 |
|  | 100 | 1821.23 | 6.97 | 58.439 |
| Ca^2+^ | 1 | 3842.44 | 9.33 | 123.294 |
|  | 10 | 5430.45 | 9.33 | 174.249 |
|  | 100 | 4223.24 | 6.94 | 135.513 |
| Na^+^ | 1 | 3116.53 | 0.03 | 100.002 |
|  | 10 | 3484.28 | 0.53 | 111.802 |
|  | 100 | 3034.75 | 2.56 | 97.377 |

Abbreviations: S.D. = Standard Deviation (n=3)

**Table 73:** Effect of various metal ions on catalytic efficiency of partially purified alkaline protease from *Bacillus pseudofirmus* LAP220

| **Metal ions** | **Concentration**  **(mM)** | **Enzyme activity (U/mL)** | **± S.D.** | **Residual activity (%)** |
| --- | --- | --- | --- | --- |
| Control | - | 3041.38 |  | 100.000 |
| Mg^2+^ | 1 | 3948.32 | 4.03 | 129.820 |
|  | 10 | 5943.98 | 2.42 | 195.437 |
|  | 100 | 5335.87 | 6.84 | 175.442 |
| Mn^2+^ | 1 | 3843.57 | 7.55 | 126.376 |
|  | 10 | 5833.35 | 5.38 | 191.799 |
|  | 100 | 1393.58 | 5.99 | 45.821 |
| Cu^2+^ | 1 | 3858.52 | 11.35 | 126.867 |
|  | 10 | 5838.65 | 15.45 | 191.974 |
|  | 100 | 1833.19 | 3.42 | 60.275 |
| Fe^3+^ | 1 | 2283.24 | 6.33 | 75.072 |
|  | 10 | 2182.24 | 9.53 | 71.752 |
|  | 100 | 1828.35 | 3.66 | 60.116 |
| Zn^2+^ | 1 | 4674.39 | 6.55 | 153.693 |
|  | 10 | 4045.25 | 5.36 | 133.007 |
|  | 100 | 1834.56 | 1.38 | 60.320 |
| Ba^2+^ | 1 | 5495.25 | 2.38 | 180.683 |
|  | 10 | 2485.69 | 3.55 | 81.729 |
|  | 100 | 193.35 | 3.03 | 6.357 |
| Hg^2+^ | 1 | 3041.48 | 0.02 | 100.003 |
|  | 10 | 1245.99 | 0.22 | 40.967 |
|  | 100 | 284.52 | 0.13 | 9.355 |
| Ca^2+^ | 1 | 3070.21 | 0.02 | 100.948 |
|  | 10 | 5965.65 | 3.45 | 196.149 |
|  | 100 | 2845.44 | 4.33 | 93.558 |
| Na^+^ | 1 | 3081.34 | 5.58 | 101.314 |
|  | 10 | 5953.75 | 8.53 | 195.758 |
|  | 100 | 1838.56 | 8.56 | 60.452 |

Abbreviations: S.D. = Standard Deviation (n=3)

**Table 74:** Effect of various metal ions on catalytic efficiency of partially purified alkaline protease from *Bacillus cohnii* LAP217

| **Metal ions** | **Concentration**  **(mM)** | **Enzyme activity (U/mL)** | **± S.D.** | **Residual activity (%)** |
| --- | --- | --- | --- | --- |
| Control | - | 4036.42 |  | 100.000 |
| Mg^2+^ | 1 | 4831.34 | 6.58 | 119.694 |
|  | 10 | 6945.32 | 6.52 | 172.066 |
|  | 100 | 3843.19 | 8.65 | 95.213 |
| Mn^2+^ | 1 | 4223.96 | 9.65 | 104.646 |
|  | 10 | 5833.52 | 3.85 | 144.522 |
|  | 100 | 2832.54 | 7.57 | 70.175 |
| Cu^2+^ | 1 | 3945.98 | 9.54 | 97.759 |
|  | 10 | 2842.46 | 13.49 | 70.420 |
|  | 100 | 1345.35 | 6.57 | 33.330 |
| Fe^3+^ | 1 | 4035.38 | 5.47 | 99.974 |
|  | 10 | 3948.38 | 6.37 | 97.819 |
|  | 100 | 2454.64 | 5.84 | 60.812 |
| Zn^2+^ | 1 | 3942.55 | 9.68 | 97.674 |
|  | 10 | 2352.48 | 3.76 | 58.281 |
|  | 100 | 675.38 | 5.98 | 16.732 |
| Ba^2+^ | 1 | 4044.38 | 6.35 | 100.197 |
|  | 10 | 5833.38 | 8.37 | 144.519 |
|  | 100 | 2845.59 | 9.25 | 70.498 |
| Hg^2+^ | 1 | 4050.25 | 11.46 | 100.343 |
|  | 10 | 3443.35 | 2.34 | 85.307 |
|  | 100 | 2832.34 | 1.47 | 70.170 |
| Ca^2+^ | 1 | 4853.37 | 9.47 | 120.239 |
|  | 10 | 5943.53 | 11.64 | 147.248 |
|  | 100 | 4985.22 | 8.57 | 123.506 |
| Na^+^ | 1 | 4036.42 | 0.05 | 100.000 |
|  | 10 | 4984.24 | 4.24 | 123.482 |
|  | 100 | 3855.23 | 7.65 | 95.511 |

Abbreviations: S.D. = Standard Deviation (n=3)

**Table 75:** Effect of various metal ions on catalytic efficiency of partially purified alkaline protease from *Brevibacterium casei* LAP223

| **Metal ions** | **Concentration**  **(mM)** | **Enzyme activity (U/mL)** | **± S.D.** | **Residual activity (%)** |
| --- | --- | --- | --- | --- |
| Control | - | 4118.42 |  | 100.000 |
| Mg^2+^ | 1 | 5734.57 | 6.47 | 139.242 |
|  | 10 | 6037.47 | 7.44 | 146.597 |
|  | 100 | 3242.34 | 9.95 | 78.728 |
| Mn^2+^ | 1 | 4118.32 | 5.36 | 99.998 |
|  | 10 | 5698.01 | 3.35 | 138.354 |
|  | 100 | 3371.28 | 9.68 | 81.859 |
| Cu^2+^ | 1 | 4119.37 | 11.36 | 100.023 |
|  | 10 | 3742.74 | 4.63 | 90.878 |
|  | 100 | 2744.58 | 5.68 | 66.642 |
| Fe^3+^ | 1 | 4118.43 | 2.25 | 100.000 |
|  | 10 | 4045.64 | 8.68 | 98.233 |
|  | 100 | 3953.35 | 5.54 | 95.992 |
| Zn^2+^ | 1 | 4190.43 | 7.94 | 101.748 |
|  | 10 | 4593.32 | 10.63 | 111.531 |
|  | 100 | 3944.85 | 11.37 | 95.786 |
| Ba^2+^ | 1 | 4593.32 | 5.78 | 111.531 |
|  | 10 | 4851.43 | 9.57 | 117.798 |
|  | 100 | 2831.29 | 4.47 | 68.747 |
| Hg^2+^ | 1 | 4043.23 | 6.68 | 98.174 |
|  | 10 | 3948.32 | 7.02 | 95.869 |
|  | 100 | 1934.76 | 5.83 | 46.978 |
| Ca^2+^ | 1 | 5843.43 | 9.61 | 141.885 |
|  | 10 | 5942.11 | 7.79 | 144.281 |
|  | 100 | 878.78 | 9.71 | 21.338 |
| Na^+^ | 1 | 4117.49 | 4.47 | 99.977 |
|  | 10 | 4075.38 | 5.53 | 98.955 |
|  | 100 | 3945.54 | 8.36 | 95.802 |

Abbreviations: S.D. = Standard Deviation (n=3)

**Table 76:** Effect of various solvents on catalytic efficiency of partially purified alkaline protease from *Halomonas venusta* LAP515

| **Solvents** | **Concentration**  **(%)** | **Enzyme**  **activity (U/mL)** | **± S.D.** |
| --- | --- | --- | --- |
| Control | - | 3006.32 |  |
| Methanol | 1 | 2874.45 | 3.74 |
|  | 10 | 2174.37 | 3.28 |
| Ethanol | 1 | 2753.64 | 2.48 |
|  | 10 | 2187.37 | 3.09 |
| Acetonitrile | 1 | 2873.73 | 1.37 |
|  | 10 | 2187.16 | 3.59 |
| Acetone | 1 | 2983.76 | 0.02 |
|  | 10 | 2173.32 | 3.45 |
| Diethyl ether | 1 | 2816.51 | 1.38 |
|  | 10 | 2200.54 | 5.42 |
| Ethyl acetate | 1 | 2182.45 | 2.57 |
|  | 10 | 1736.47 | 5.48 |

S.D. = Standard Deviation (n=3)

**Table 77:** Effect of various solvents on catalytic efficiency of partially purified alkaline protease from *Brachybacterium* sp. LAP214

| **Solvents** | **Concentration**  **(%)** | **Enzyme**  **activity (U/mL)** | **± S.D.** |
| --- | --- | --- | --- |
| Control | - | 3116.48 |  |
| Methanol | 1 | 2991.34 | 7.94 |
|  | 10 | 2183.38 | 5.44 |
| Ethanol | 1 | 3084.22 | 1.19 |
|  | 10 | 2842.27 | 4.82 |
| Acetonitrile | 1 | 2734.28 | 7.47 |
|  | 10 | 2198.38 | 1.27 |
| Acetone | 1 | 2743.21 | 3.85 |
|  | 10 | 2099.42 | 4.38 |
| Diethyl ether | 1 | 2998.37 | 3.25 |
|  | 10 | 2754.08 | 6.55 |
| Ethyl acetate | 1 | 2274.35 | 3.38 |
|  | 10 | 2093.26 | 2.43 |

S.D. = Standard Deviation (n=3)

**Table 78:** Effect of various solvents on catalytic efficiency of partially purified alkaline protease from *Bacillus pseudofirmus* LAP220

| **Solvents** | **Concentration**  **(%)** | **Enzyme**  **activity (U/mL)** | **± S.D.** |
| --- | --- | --- | --- |
| Control | - | 3041.38 |  |
| Methanol | 1 | 2718.49 | 4.83 |
|  | 10 | 2094.32 | 7.28 |
| Ethanol | 1 | 2671.39 | 9.16 |
|  | 10 | 2197.03 | 4.73 |
| Acetonitrile | 1 | 2617.39 | 7.83 |
|  | 10 | 2064.04 | 2.39 |
| Acetone | 1 | 2466.16 | 9.16 |
|  | 10 | 2163.37 | 3.56 |
| Diethyl ether | 1 | 3011.36 | 8.04 |
|  | 10 | 2973.17 | 2.45 |
| Ethyl acetate | 1 | 2367.51 | 5.34 |
|  | 10 | 2028.36 | 5.22 |

S.D. = Standard Deviation (n=3)

**Table 79:** Effect of various solvents on catalytic efficiency of partially purified alkaline protease from *Bacillus cohnii* LAP217

| **Solvents** | **Concentration**  **(%)** | **Enzyme**  **activity (U/mL)** | **± S.D.** |
| --- | --- | --- | --- |
| Control | - | 4036.42 |  |
| Methanol | 1 | 3283.26 | 3.85 |
|  | 10 | 2984.27 | 7.68 |
| Ethanol | 1 | 3764.22 | 4.39 |
|  | 10 | 3093.02 | 9.55 |
| Acetonitrile | 1 | 3732.19 | 2.11 |
|  | 10 | 2771.21 | 3.04 |
| Acetone | 1 | 4002.47 | 5.02 |
|  | 10 | 3988.31 | 6.03 |
| Diethyl ether | 1 | 3230.32 | 5.43 |
|  | 10 | 3109.55 | 2.11 |
| Ethyl acetate | 1 | 3350.54 | 3.53 |
|  | 10 | 3099.8 | 7.39 |

S.D. = Standard Deviation (n=3)

**Table 80:** Effect of various solvents on catalytic efficiency of partially purified alkaline protease from *Brevibacterium casei* LAP223

| **Solvents** | **Concentration**  **(%)** | **Enzyme**  **activity (U/mL)** | **± S.D.** |
| --- | --- | --- | --- |
| Control | - | 4118.42 |  |
| Methanol | 1 | 3988.46 | 4.37 |
|  | 10 | 1373.36 | 2.18 |
| Ethanol | 1 | 3655.47 | 5.49 |
|  | 10 | 2633.34 | 9.04 |
| Acetonitrile | 1 | 2637.48 | 2.44 |
|  | 10 | 1773.38 | 3.19 |
| Acetone | 1 | 3116.23 | 7.39 |
|  | 10 | 1983.28 | 2.34 |
| Diethyl ether | 1 | 3784.32 | 5.4 |
|  | 10 | 2983.26 | 9.54 |
| Ethyl acetate | 1 | 3562.09 | 3.48 |
|  | 10 | 2092.28 | 1.38 |

S.D. = Standard Deviation (n=3)

**Table 81:** Effect of various commercial detergents on catalytic efficiency of partially purified alkaline protease from *Halomonas venusta* LAP515

| **Detergents** | **Concentration**  **(mg/mL)** | **Enzyme**  **activity (U/mL)** | **± S.D.** | **Residual activity (%)** |
| --- | --- | --- | --- | --- |
| Control | - | 3006.32 | 2.74 | 100.000 |
| Ariel™ | 7 | 2938.02 | 1.28 | 97.728 |
|  | 14 | 2382.22 | 1.39 | 79.240 |
|  | 21 | 2232.29 | 5.40 | 74.253 |
| Surf excel™ | 7 | 2738.02 | 3.59 | 91.075 |
|  | 14 | 2328.39 | 5.49 | 77.450 |
|  | 21 | 2028.32 | 0.03 | 67.469 |
| Nirma™ | 7 | 2736.27 | 1.23 | 91.017 |
|  | 14 | 2510.09 | 4.63 | 83.494 |
|  | 21 | 2303.28 | 1.39 | 76.615 |
| Sasa™ | 7 | 2808.28 | 3.48 | 93.413 |
|  | 14 | 2493.292 | 8.56 | 82.935 |
|  | 21 | 2200.09 | 1.00 | 73.182 |
| Wheel™ | 7 | 2804.41 | 5.30 | 93.284 |
|  | 14 | 2783.85 | 2.49 | 92.600 |
|  | 21 | 2550.98 | 5.38 | 84.854 |
| Rin™ | 7 | 2451.75 | 2.48 | 81.553 |
|  | 14 | 2356.09 | 5.38 | 78.371 |
|  | 21 | 2240.12 | 1.23 | 74.514 |
| Tide™ | 7 | 2854.03 | 2.37 | 94.934 |
|  | 14 | 2730.73 | 5.58 | 90.833 |
|  | 21 | 2609.34 | 1.38 | 86.795 |

S.D. = Standard Deviation (n=3)

**Table 82:** Effect of various commercial detergents on catalytic efficiency of partially purified alkaline protease from *Brachybacterium* sp. LAP214

| **Detergents** | **Concentration**  **(mg/mL)** | **Enzyme**  **activity (U/mL)** | **± S.D.** | **Residual activity (%)** |
| --- | --- | --- | --- | --- |
| Control | - | 3116.48 |  | 100.000 |
| Ariel™ | 7 | 3048.74 | 2.38 | 97.826 |
|  | 14 | 2912.24 | 1.39 | 93.446 |
|  | 21 | 2132.48 | 5.54 | 68.426 |
| Surf excel™ | 7 | 3002.34 | 2.48 | 96.338 |
|  | 14 | 2913.37 | 5.38 | 93.483 |
|  | 21 | 2123.26 | 2.48 | 68.130 |
| Nirma™ | 7 | 3093.42 | 0.38 | 99.260 |
|  | 14 | 2532.48 | 4.39 | 81.261 |
|  | 21 | 2123.03 | 1.38 | 68.123 |
| Sasa™ | 7 | 2737.42 | 4.49 | 87.837 |
|  | 14 | 2372.11 | 6.58 | 76.115 |
|  | 21 | 2162.37 | 2.49 | 69.385 |
| Wheel™ | 7 | 2837.43 | 0.34 | 91.046 |
|  | 14 | 2373.26 | 3.42 | 76.152 |
|  | 21 | 2126.26 | 1.38 | 68.226 |
| Rin™ | 7 | 2833.37 | 5.59 | 90.916 |
|  | 14 | 2773.25 | 2.48 | 88.987 |
|  | 21 | 2171.36 | 1.38 | 69.673 |
| Tide™ | 7 | 2234.27 | 2.48 | 71.692 |
|  | 14 | 2037.27 | 3.33 | 65.371 |
|  | 21 | 1813.37 | 1.23 | 58.186 |

S.D. = Standard Deviation (n=3)

**Table 83:** Effect of various commercial detergents on catalytic efficiency of partially purified alkaline protease from *Bacillus pseudofirmus* LAP220

| **Detergents** | **Concentration**  **(mg/mL)** | **Enzyme**  **activity (U/mL)** | **± S.D.** | **Residual activity (%)** |
| --- | --- | --- | --- | --- |
| Control | - | 3041.38 |  | 100 |
| Ariel™ | 7 | 2931.27 | 1.30 | 96.380 |
|  | 14 | 2735.63 | 2.23 | 89.947 |
|  | 21 | 2632.16 | 3.39 | 86.545 |
| Surf excel™ | 7 | 2914.37 | 0.54 | 95.824 |
|  | 14 | 2742.56 | 2.42 | 90.175 |
|  | 21 | 2694.47 | 0.36 | 88.594 |
| Nirma™ | 7 | 2742.17 | 1.78 | 90.162 |
|  | 14 | 2274.47 | 0.03 | 74.784 |
|  | 21 | 2173.43 | 2.27 | 71.462 |
| Sasa™ | 7 | 2842.43 | 2.84 | 93.459 |
|  | 14 | 2634.15 | 8.37 | 86.610 |
|  | 21 | 2135.36 | 2.37 | 70.210 |
| Wheel™ | 7 | 2845.25 | 0.63 | 93.551 |
|  | 14 | 2174.38 | 1.32 | 71.493 |
|  | 21 | 2023.42 | 2.63 | 66.530 |
| Rin™ | 7 | 2835.42 | 2.347 | 93.228 |
|  | 14 | 2640.14 | 1.18 | 86.807 |
|  | 21 | 2448.36 | 0.21 | 80.502 |
| Tide™ | 7 | 2944.17 | 2.29 | 96.804 |
|  | 14 | 2743.57 | 1.22 | 90.208 |
|  | 21 | 2645.21 | 2.39 | 86.974 |

S.D. = Standard Deviation (n=3)

**Table 84:** Effect of various commercial detergents on catalytic efficiency of partially purified alkaline protease from *Bacillus cohnii* LAP217

| **Detergents** | **Concentration**  **(mg/mL)** | **Enzyme**  **activity (U/mL)** | **± S.D.** | **Residual activity (%)** |
| --- | --- | --- | --- | --- |
| Control | - | 4036.42 |  | 100.000 |
| Ariel™ | 7 | 3842.47 | 3.34 | 95.195 |
|  | 14 | 3193.43 | 1.39 | 79.115 |
|  | 21 | 3049.24 | 1.09 | 75.543 |
| Surf excel™ | 7 | 3811.04 | 0.32 | 94.416 |
|  | 14 | 3741.35 | 0.00 | 92.690 |
|  | 21 | 3182.37 | 0.65 | 78.841 |
| Nirma™ | 7 | 3754.18 | 1.38 | 93.008 |
|  | 14 | 3653.18 | 2.47 | 90.505 |
|  | 21 | 3183.09 | 1.39 | 78.859 |
| Sasa™ | 7 | 3743.17 | 4.48 | 92.735 |
|  | 14 | 3654.32 | 2.44 | 90.534 |
|  | 21 | 3038.02 | 1.38 | 75.265 |
| Wheel™ | 7 | 3842.18 | 2.45 | 95.188 |
|  | 14 | 3224.31 | 2.48 | 79.880 |
|  | 21 | 3002.18 | 1.23 | 74.377 |
| Rin™ | 7 | 3842.18 | 2.00 | 95.188 |
|  | 14 | 3228.47 | 2.47 | 79.984 |
|  | 21 | 2849.37 | 1.39 | 70.592 |
| Tide™ | 7 | 3734.15 | 1.38 | 92.511 |
|  | 14 | 3647.32 | 3.03 | 90.360 |
|  | 21 | 2349.48 | 2.20 | 58.207 |

S.D. = Standard Deviation (n=3)

**Table 85:** Effect of various commercial detergents on catalytic efficiency of partially purified alkaline protease from *Brevibacterium casei* LAP223

| **Detergents** | **Concentration**  **(mg/mL)** | **Enzyme**  **activity (U/mL)** | **± S.D.** | **Residual activity (%)** |
| --- | --- | --- | --- | --- |
| Control | - | 4118.42 |  | 100 |
| Ariel™ | 7 | 3948.04 | 1.38 | 95.863 |
|  | 14 | 3724.24 | 2.48 | 90.429 |
|  | 21 | 3228.43 | 4.40 | 78.390 |
| Surf excel™ | 7 | 3738.29 | 2.29 | 90.770 |
|  | 14 | 3448.28 | 1.19 | 83.728 |
|  | 21 | 3138.27 | 4.48 | 76.201 |
| Nirma™ | 7 | 3940.95 | 3.58 | 95.691 |
|  | 14 | 3845.24 | 2.40 | 93.367 |
|  | 21 | 3056.64 | 8.42 | 74.219 |
| Sasa™ | 7 | 3484.22 | 9.47 | 84.601 |
|  | 14 | 3299.25 | 3.38 | 80.110 |
|  | 21 | 2980.22 | 1.38 | 72.363 |
| Wheel™ | 7 | 3911.72 | 3.38 | 94.981 |
|  | 14 | 3839.03 | 3.31 | 93.216 |
|  | 21 | 3002.17 | 4.42 | 72.896 |
| Rin™ | 7 | 3493.72 | 2.29 | 84.832 |
|  | 14 | 3092.38 | 2.34 | 75.087 |
|  | 21 | 2738.28 | 2.11 | 66.489 |
| Tide™ | 7 | 3748.28 | 1.38 | 91.013 |
|  | 14 | 3732.18 | 2.38 | 90.622 |
|  | 21 | 3117.27 | 2.11 | 75.691 |

S.D. = Standard Deviation (n=3)
